# Stability profiling of anti-malarial drug piperaquine phosphate and impurities by HPLC-UV, TOF-MS, ESI-MS and NMR

**DOI:** 10.1186/1475-2875-13-401

**Published:** 2014-10-13

**Authors:** Fang Yan, Jie Liu, Xuefang Zeng, Yuan Zhang, Taijun Hang

**Affiliations:** Department of Pharmacy, China Pharmaceutical University, Tongjiaxiang 24, Nanjing, P R China; Hainan Provincial Institute for Drug and Food Control, Longhua Road 8, Haikou, P R China; Suzhou institute for food and drug control, Wuzhong Road 1336, Suzhou, RP China

**Keywords:** Anti-malarial, Piperaquine, Related impurities, Stability profile, RP-HPLC, TOF-MS, ESI-MS, NMR

## Abstract

**Background:**

Piperaquine, 1,3-bis-[4-(7-chloroquinolyl-4)-piperazinyl-1]-propane, is an anti-malarial compound belonging to the 4-aminoquinolines, which has received renewed interest in treatment of drug resistant falciparum malaria in artemisinin-based combination therapy with dihydroartemisinin. The impurity profile of this drug product is paid an ever-increasing attention. However, there were few published studies of the complete characterization of related products or impurities in piperaquine phosphate bulk and forced degradation samples.

**Methods:**

The impurities in piperaquine phosphate bulk drug substance were detected by a newly developed gradient phase HPLC method and identified by TOF-MS and ESI-MS. The structures of impurities were confirmed by NMR. Forced degradation studies were also performed for the stability of piperaquine phosphate bulk drug samples and the specificity of the newly developed HPLC method. *In silico* toxicological predictions for these piperaquine phosphate related impurities were made by Toxtree^®^ and Derek^®^.

**Results:**

Twelve impurities (imp-1**–**12) were detected and identified, of which eight impurities (imp-1, 2, 4, 6–10) were first proposed as new related substances. Based on TOF-MS/ESI-MS and NMR analysis, the structures of imp-2, 6 and 12 were characterized by their synthesis and preparation. The possible mechanisms for the formation of impurities were also discussed. These piperaquine phosphate related impurities were predicted to have a toxicity risk by Toxtree^®^ and Derek^®^.

**Conclusions:**

From forced degradation and bulk samples of piperaquine phosphate, twelve compounds were detected and identified to be piperaquine phosphate related impurities. Two of the new piperaquine phosphate related substances, imp-2 and imp-6, were identified and characterized as 4-hydroxy-7-chloro-quinoline and a piperaquine oxygenate with a piperazine ring of nitrogen oxide in bulk drug and oxidation sample, respectively. The MS data of imp-1, 2, 4, 6–10 were first reported. The *in-silico* toxicological prediction showed a toxicity risk for piperaquine related impurities by Toxtree^®^ and Derek^®^.

**Electronic supplementary material:**

The online version of this article (doi:10.1186/1475-2875-13-401) contains supplementary material, which is available to authorized users.

## Background

Malaria, caused by the mosquito-borne protozoan parasite *Plasmodium falciparum*, is one of the major global public health challenges with an estimated 219 million clinical cases and 655,000 in 2010, mainly in children aged less than five years old from sub-Saharan Africa [1, 2]. Piperaquine, 1,3-bis-[4-(7-chloroquinolyl-4)-piperazinyl-1]-propane, is an anti-malarial compound belonging to the 4-aminoquinolines, which was first synthesized as compound 13228 RP by Rhone Poulenc in France in the 1950s. Piperaquine was rediscovered by Shanghai Research Institute of Pharmaceutical Industry in the 1960s and in 1970s, and rapidly replaced chloroquine as first-line monotherapy in southern China [3], until resistance emerged in the 1980s.

Recently piperaquine has received renewed interest in treatment of drug resistant falciparum malaria, as it has proved to be a suitable partner drug in artemisinin-based combination therapy (ACT) to improve anti-malarial effectiveness and to keep the selection of drug-resistant parasites to minimum [4]. Meanwhile, it is now commercially available in fixed combination products, mostly with dihydroartemisinin, which are proved to be highly efficacious for treatment of uncomplicated falciparum malaria [5, 6]. The anti-malarial therapeutic efficacy studies conducted in Cambodia India and Vietnam, showed that for *Plasmodium* vivax and *P. falciparum* infection the therapeutic efficacy of the treatment of dihydroartemisinin-piperaquine remained high (100%) as an appropriate new first-line treatment [6–8]. In China, the piperaquine tablets were recommended at the dose of 600 mg monthly to prevent malaria. In the toxicological and clinic research, the most common adverse effects of piperaquine are dizziness, headache and gastrointestinal symptoms (nausea, vomiting, diarrhoea and abdominal pain) with less frequent side effects including urticaria, elevated serum alanine aminotransferase (ALT), low serum glucose, prolonged electrocardiographic QT interval and decreased white cell count [9–11]. An evaluation of piperaquine for reproduction did not show any genotoxic or clastogenic potential. No evidence of adverse effects on pregnancy in humans and animals has been observed [12, 13]. The piperaquine combination exerted a significant treatment and post-treatment prophylactic effects, indicating that piperaquine is a new partner drug of ACT displaying high efficacy and safety in the treatment of malaria.

The quality of a drug product is related not only with the contents of the active drug substances, but also with its impurities. Though there is an ever-increasing attention on impurity profile, just Chinese Pharmacopeia has already established specification limits for the total related impurities of piperaquine phosphate by HPLC-UV method [14]. Zhang *et al.* [15] developed an LC method for the analysis of piperaquine phosphate and related substances with the impurities limit of 0.21% - 0.39% under photo degradation condition. Now, it is mandatory to identify and characterize the impurities in the pharmaceutical product, if present above the accepted limits of 0.1% [16]. Dongre *et al.* [17] detected and identified only four impurities, such as 7-chloro-4-piperazinyl quinoline, 1-chloro-3-(7-chloro-4-quinolyl-4-piperazinyl) propane, 1-(1-5-chloro-4-quinolyl-4-piperazinyl)-3-(1-7-chloro-4-quinolyl-4-piperazinyl) propane and 1,4-bis-(4,7-dichloroquinoline) piperazine in piperaquine phosphate bulk drug substance by gradient reverse phase high performance liquid chromatographic (HPLC) and LC/MS/MS methods. The structures of three impurities were synthesized and confirmed by NMR and IR. Although these studies based on HPLC, LC-MS/MS and spectroscopic methods were reported in the literature for the quality and quantitative analysis of piperaquine phosphate and its impurities [14, 15, 17], there is few published studies of the complete characterization of related products or impurities in piperaquine phosphate as active pharmaceutical ingredient.

In this study, twelve potential impurities were detected, including new degradants, in piperaquine phosphate bulk sample using a newly developed gradient reversed phase HPLC methods. A comprehensive study was undertaken for the identification of these impurities by LC-TOF/MS and ESI-MS followed by their synthesis and further characterization by NMR. The *in silico* toxicological evaluation of these impurities was exerted by Toxtree^®^ and Derek^®^.

## Methods

### Samples and chemicals

The reference standards of piperaquine phosphate (purity > 99.0%), piperaquine phosphate bulk samples, 4-hydroxy-7-chloro-quinoline (**imp-2**) (purity > 99.0%) and 1, 4-bis-(4, 7-dichloroquinoline) piperazine (**imp-12**) (purity > 99.0%) were kindly provided by Chongqing Southwest No.2 Pharmaceutical Factory Co., LTD. 1, 3-bis [1, 4-(4,7-dichloroquinoline) piperazin] propane nitrogen oxides (**imp-6**) (purity > 98.0%) was prepared by HPLC in our laboratory from piperaquine phosphate forced degradation samples. Acetonitrile of HPLC grade was used for the analytical HPLC analysis, and purchased from Merk, Darmstadt, Germany. Deionized water (18 MΩ cm) was prepared with a Millipore Milli Q-Plus purification system (Millipore Corp., MA, USA). Ammonium acetate and ammonia water were of analytical reagent grade and purchased from Sigma-Aldrich (St Louis, USA). Other reagents were analytical reagent grade and purchased from Nanjing Chemical Co. (Nanjing, China). The NMR solvent of dimethyl sulphoxide-*d*_6_ and tridecafluoroheptanoic acid-*d* were purchased from Merk, Darmstadt, Germany.

### Chramatographic conditions

The HPLC-PDA chromatographic system consisting of a Hitachi Chromaster separation module and a Hitachi Chromaster photodiode array detector (Hitachi, Tokyo, Japan) was used for analytical and preparative separations with Empower 3.0 software (Milford, MA, USA). HPLC separation was performed on a Phecda C18 analytical column (250 mm × 4.6 mm, 5 μm) at temperature of 30°C. The detection wavelength was set at 317 nm. The gradient elution at the flow rate of 1.0 mL/min was employed with acetonitrile as mobile phase A and 0.1% ammonium acetate solution (pH adjusted to 7.0 by using ammonia water) as mobile phase B, with the gradient programme of time (min)/% A: 0/40, 5/40, 54/100, 54.1/40, 60/40. The sample injection volume was 20 μL.

### TOF-MS and ESI-MS conditions

Accurate mass measurements were performed on an Agilent 6224 accurate-mass time-of-flight (TOF) mass spectrometer with qualitative Analysis B.04.00 software (All Agilent Technologies, Santa Clara, CA, USA). The operating parameters in the positive ion detection mode were as follows: drying gas (N_2_) flow rate, 10.0 L/min; sheath gas temperature, 350°C; capillary, +4000 V; fragmentor, 135 V; skimmer,; collision energy,; and mass range, 100–1500 Da. The ESI-MS/MS spectra was carried out by Thermo-Finnigan TSQ Quantum Ultra tandem mass spectrometer equipped equiped with an electrospray ionization source (ESI), and Xcalibur 1.4 software was used for data acquisition and processing (All Thermo-Finnigan, San Jose, CA, USA). The mass spectra of ESI-MS/MS were recorded in the same ion detection mode to analysis the fragment ions of the related substances. The spray voltage was set at 5000 V. The heated capillary temperature was 350°C. The sheath gas and the auxiliary gas were set at 45 and 10 psi, respectively. The fragment ions were produced by collision-induced dissociation of the selected precursor ions with the collision energy of 35 eV. In the LC-MS/MS measurements chromatographic conditions described in the section of “Chromatographic conditions” were used.

### ^1^H and ^13^C NMR spectroscopy

The ^1^H, ^13^C NMR spectra of the impurities was performed on Bruker AVANCE DRX-500 spectrometer using dimethyl sulphoxide-*d*_6_ as solvent and tetramethylsilane (TMS) as internal standard.

### Sample preparation

#### Preparation of forced degradation samples

Forced degradation studies can identify the degradation products, establish the degradation pathways and find the intrinsic stability of the molecule. The preparation of all forced degradation samples were conducted by stressing with acid (0.1 M HCl, 60°C, 30 min), alkaline (0.1 M NaOH, 60°C, 30 min), hydrolysis and oxidation (30% H_2_O_2_, room temperature, 30 min) and photo (UV light and cool white fluorescent, 10 days) according to option 2 of Q1B in ICH guidelines. The degraded samples were neutralized (for acidic and basic hydrolysed) and diluted to final concentration of 800 μg/mL before the assay of the degradation impurities.

#### Enrichment of impurity-6 in oxidation samples

The isolation and enrichment of **imp-6** was performed on a Shimadzu LC-2010 HT Liquid Chromatograph equipped with a SPD-10AVP UV–vis detector (Shimadzu Corp., Kyoto, Japan). The Chromatographic conditions were performed according to the section of “Chromatographic conditions”. The test solution of oxidation samples (30% H_2_O_2_, room temperature, 12 h) was prepared at the final concentration of 300 μg/mL. The **imp-6** solution was repeatedly collected at the retention time region of 5.8-6.3 min, before evaporated to dryness under high vaccum. The residue of **imp-6** was obtained with the purity above 98% based on HPLC analysis by area normalization method.

#### *In silico*toxicological predictions

The structure-activity relationships ((Q)SAR), based on the concept that chemical structure determines the biological activity of a molecule, are employed as scientifically credible tools for predicting the acute toxicity of chemicals relevant to public and animal health [18]. Recently, these (Q)SAR programmes will play an important role in future chemical policies, such as in the European Union and the Netherlands, to reduce animal testing and costs and to speed up the number of risk assessments for hazardous chemicals. To evaluate the toxicological characters of piperaquine related impurities *in silico*, Toxtree^®^ (v.1.60, Ideaconsult Ltd., Sofia, Bulgaria) and Derek^®^ (Nexus v3.0.1, Lhasa Limited, Leeds, UK) were selected from different two sources of toxicity predictions [19, 20]. Toxtree^®^ is a full-featured and flexible user-friendly open source application, which is able to estimate toxic hazard by applying a decision tree approach and making (Q)SAR-based predictions for a number of toxicological endpoints by different modules. Three Toxtree^®^ modules, such as Cramer rules with extension, Bengni/Bossa rulebase for mutagenicity and carcinogenicity and structure alerts for the *in vivo* micronucleus assay in rodents, were used to generate hazard estimations. Derek^®^ for windows, a knowledge-based expert system, predicts the toxicity of a compound from its chemical structural alerts, rules and examples, which describes relationship between a structural feature (toxicophore) and its associated toxicity. A broad range of toxicological endpoints are covered, including carcinogenicity, genotoxicity, hepatotoxicity, HERG channel inhibition, reproductive toxicity and skin sensitization.

## Results and discussion

### Detection of impurities by HPLC-UV/DAD

**Figure 1:**
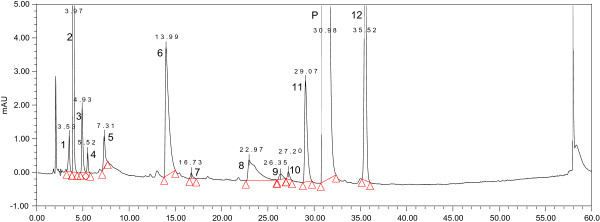
**UV chromatogram of piperaquine crude sample.** The imp-1, 2, 4 (1, 2, 4) were the starting material. The imp-3, 5, 11, 12 (3, 5, 11, 12) were the by-product in the synthetic reaction of piperaquine phosphate (P). The imp-6, 7, 8, 10 (6, 7, 8, 10) were the oxidation products and the imp-9 (9) was the degradation product of piperaquine phosphate.

The main aim of this study was to develop a selective and sensitive method for analysis of piperaquine and its related substances originated from the synthesis and forced degradation. According to the reported methods for the analysis of piperaquine [14, 15, 17], different types of commercial C_18_ columns were tested for their selectivity toward the impurities and piperaquine. Finally, a Phecda-C_18_ (250 mm × 4.6 mm, 5 μm) column was selected. A volatile mobile phase was prerequisite for the analysis of LC-MS. However, in most of the previous method [14, 15], the mobile phases contained non-volatile substances, including phosphate buffers and phosphate. Although Dongre *et al.* [17] reported the analysis method of volatile mobile phases, it showed the poor selectivity of twelve impurities. Therefore, in our study, several mobile phases consisting of different volatile buffers and organic modifiers were tried with various gradient elution. The solution of 0.1% ammonium acetate with pH of 7.0 was more suitable than 0.01 M ammonium acetate or 0.2% formic acid for baseline separation and symmetrical peaks, in combination with acetonitrile as organic modifier (detailed in Additional file 1). Optimized chromatographic conditions were described in the section of “Chromatographic conditions” and the typical chromatogram of a bulk sample was shown in Figure 1 (the typical chromatograms of the samples from other API suppliers shown in Additional file 2). The peak of piperaquine was completely separated from all of twelve impurities in 60 minutes.

Forced degradation experiment was performed by exposing piperaquine phosphate bulk to diverse stress conditions for different periods. Degradation was not observed in piperaquine forced samples subjected to photo and acid. Significant degradation of the drug substance and product was detected under basic and oxidative forced conditions, leading to the formation of **imp-6**–**10**. Peak purity test results derived from DAD detector, confirmed that the piperaquine peak and all of the impurity peaks (**imp-1**–**12**) were homogeneous and pure in all of the forced and bulk samples.

### Structure elucidation of related impurities by HPLC-ESI-MS/TOF-MS

**Figure 2:**
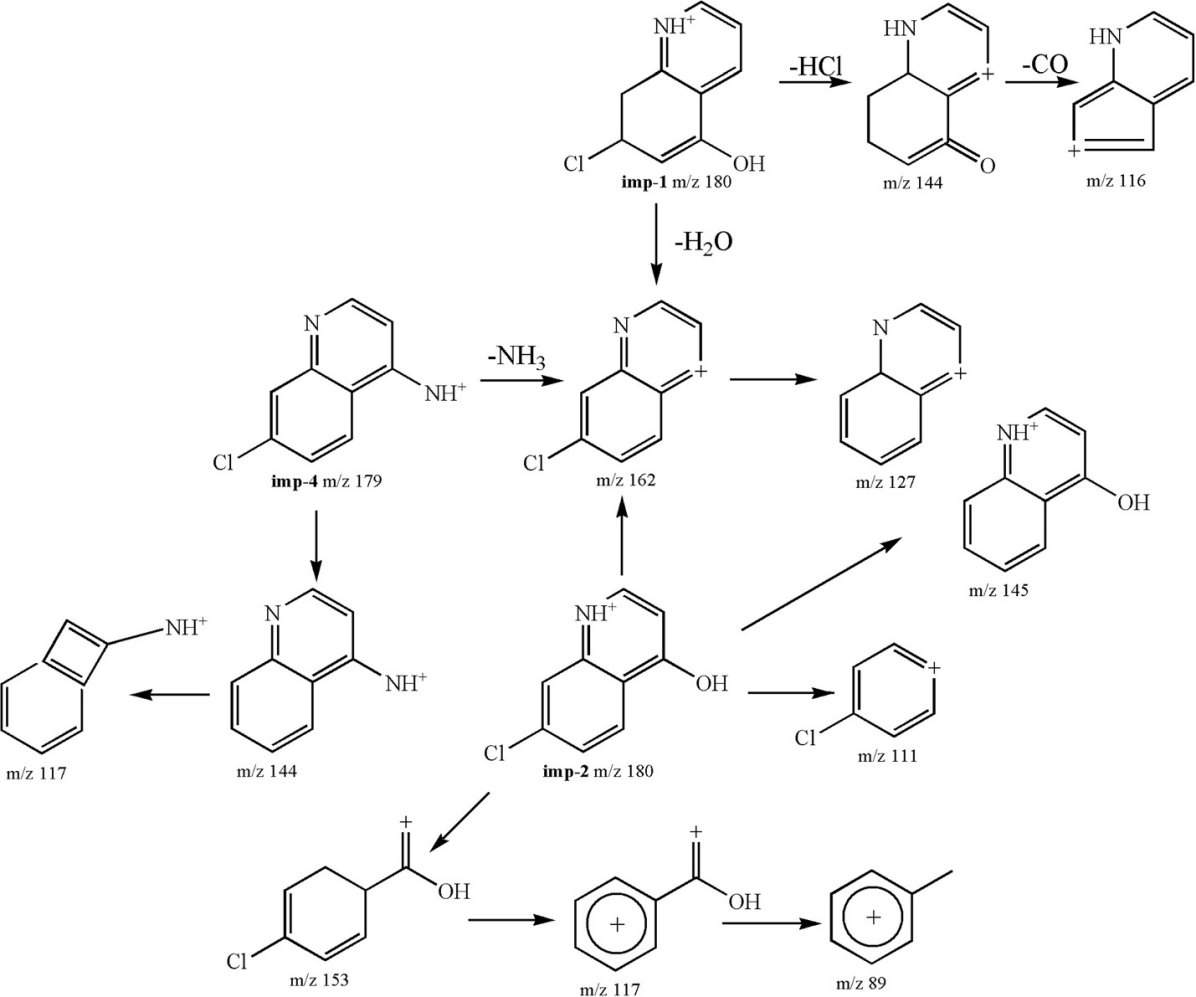
**Probable fragmentation pathways of imp-1, 2 and 4.**

**Figure 3:**
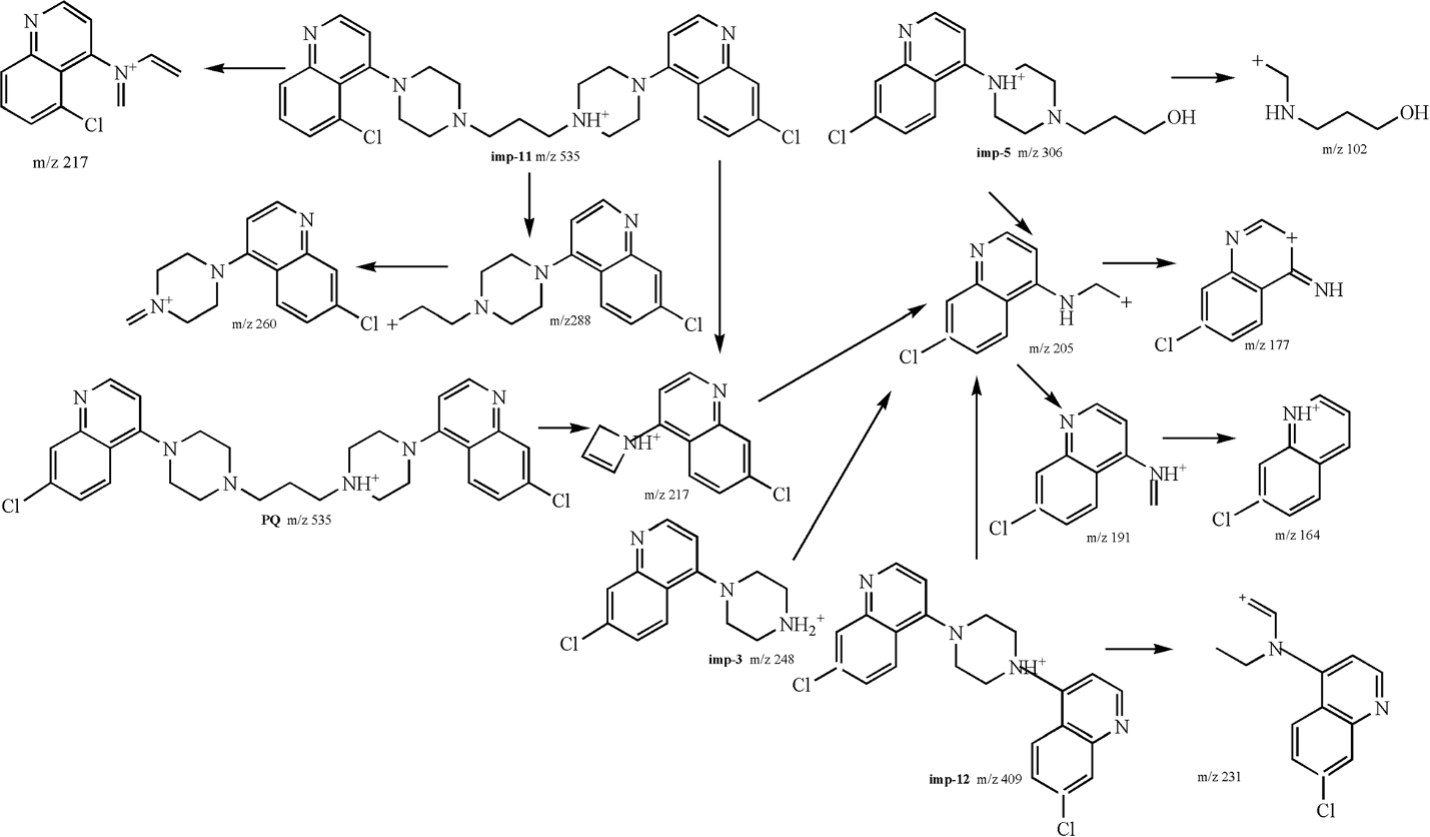
**Probable fragmentation pathways of piperaquine and imp-3, 5, 11, 12.**

**Figure 4:**
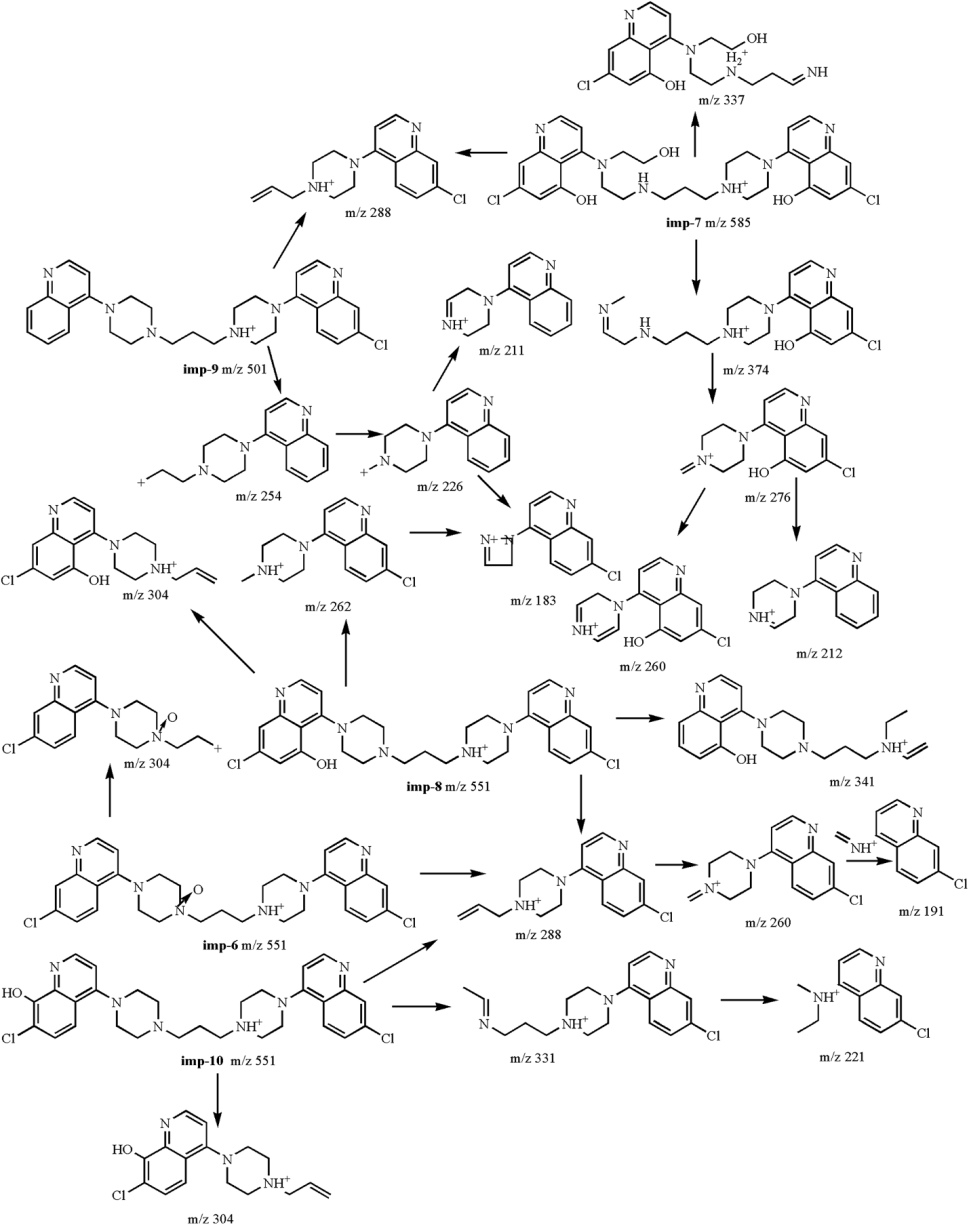
**Probable fragmentation pathways of imp-6, 7, 8, 9 and 10.**

**Figure 5:**
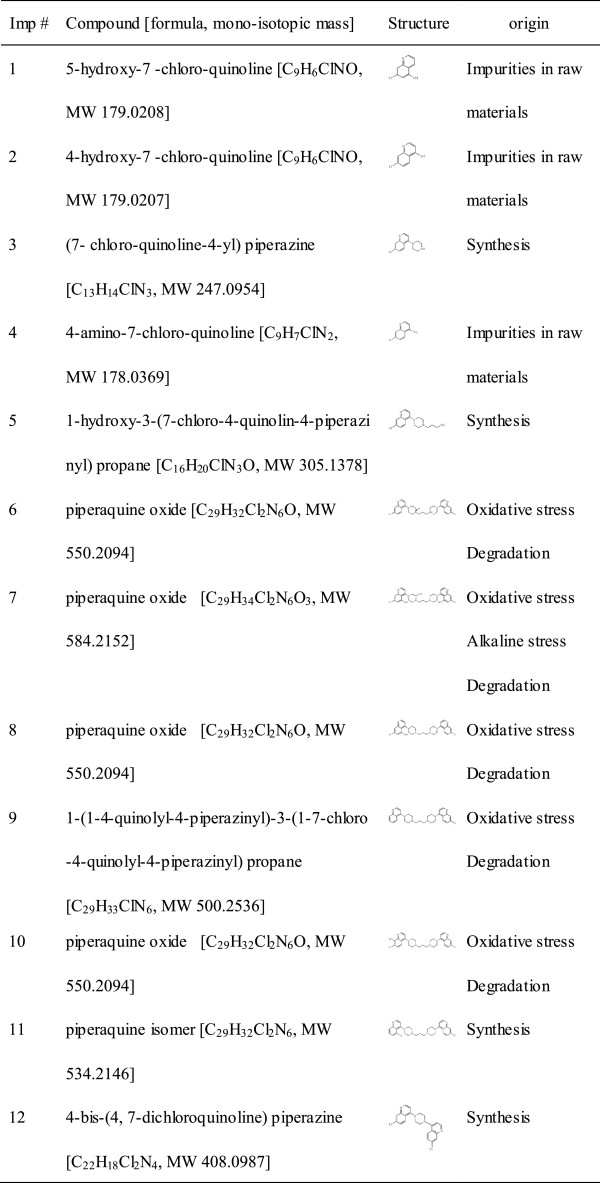
**Structural information of the piperaquine phosphate related impurities.**

All the MS and MS^n^ spectrum of piperaquine and related impurities were obtained by the method described in the section of “TOF-MS and ESI-MS conditions”. Mass spectra were recorded in positive ion mode (the mass spectra of piperaquine and related impurities shown in Additional files 3, 4, 5, 6, 7, 8, 9, 10, 11, 12, 13, 14 and 15). In order to understand the mass spectral behaviour of related impurities, a detailed study of the fragmentation pattern of the main drug component was carried on. The accurate pseudomolecular ion peak [M + H]^+^ of piperaquine, measured by Q-TOF instrument was 535.2142 Da, in agreement with the reported data (C_29_H_32_Cl_2_N_6_, MW = 534.2065) [17]. In the MS^n^ spectrum, piperaquine produced fragmentation ion at m/z 288 by the loss of 4-(7-chloro- quinoline-4-yl) piperazine. The ion peaks at m/z 260 (-28 Da), 205 (-83 Da) and 217 (-71 Da) can be attributed to the loss of -C_2_H_5_ group and the rearrangement of ring-opening, respectively. The minor ion at 164 was generated by the loss of -C_2_H_5_ group from the ion at m/z 205. The additional fragment ion at 164 yielded low abundant ion at 217, by the loss of four-numbered ring group. The main fragmentation pattern was in agreement with the literature data [17], and the probable fragmentation pathway of piperaquine was shown in Figures 2, 3 and 4. The TOF-MS and ESI-MS data and proposed chemical structure of related impurities in bulk drug and forced degradation samples were shown in Figure 5.

The **imp-1** and the **imp-2** (Figure 2), with accurate positive ions at m/z 180.0208 and 180.0207, had the same molecular formula for pseudomolecular ion (C_9_H_7_ClNO^+^). In the MS^n^ spectrum of **imp-2**, the main fragment ions at 145, 117, 111 and 89 were attributed to the structure of 7-chloro-quinoline. According to the synthetic route of piperaquine and the literature, the probable structure of **imp-2** could be 4-hydroxy-7-chloro-quinoline. The pseudomolecular ion of **imp-1** at m/z 180 produced ions at m/z 162 (-H_2_O) and 144 (-HCl). Further fragmentation of the ion at m/z 144 yielded an ion at m/z 116 by the loss of -CO group, which indicated that a hydroxyl group substituted at a different position of **imp-2**. Thus, the proposed structure of **imp-1** could be 5-hydroxy-7 -chloro-quinoline. The **imp-4** (Figure 2) with the accurate protonated ion at m/z 179.0369, yielded the ion at 144 with the same ion of the **imp-1** at 162, by the loss of -NH_3_ group (-17 Da) and chlorine radical (-35 Da), respectively. Furthermore the fragment ion at m/z 162 produced the ion at m/z 127 by the loss of chlorine radical. The fragmentation of ion at mz/117 was attributed to the ion at m/z 144 by the rearrangement of quinoline ring-opening from six- to four-numbered ring, which was consistent with the structure of (7-chloro-quinoline-4-yl) piperazine. Thus, the proposed structure of **imp-4** could be 4-amino-7-chloro-quinoline. The probable fragmentation pathways of **imp-1**, **imp-2** and **imp-4** were shown in Figure 2.

The **imp-3** (Figure 3) with the accurate [M + H]^+^ ion at m/z 248.0954, produced the abundant ion at m/z 205 by ring-opening (-43 Da). Further fragmentation of the ion at m/z 205 yielded ions at m/z 191 and 177 by the rearrangement (Figure 3). According to the main fragment ions at m/z 205, 191 and 177, it was inferred that the probable structure of **imp-3** could be (7- chloro-quinoline-4-yl) piperazine. The **imp-5** (Figure 3) with the accurate [M + H]^+^ ion at m/z 306.1378, produced the same fragmentation pattern as **imp-3** with the abundant ions at m/z 205, 191 and 164, which indicated that the structure of the **imp-5** also could contain (7-chloro-quinoline-4-yl) piperazine. The presence of minor ion at m/z 102, which was formed by piperazine ring-opening (-204 Da), indicated that the probable structure of **imp-5** could be 1-hydroxy-3-(7-chloro-4-quinolin-4-piperazinyl) propane. The **imp-12** (Figure 3) had the accurate [M + H]^+^ ion at m/z 409.0987, with the same main fragment ions as **imp-3** and **imp-5** at m/z 205, 177 and 164, which suggested that the structure of **imp-12** could also contain (7-chloro-quinoline-4-yl) piperazine. Considering the published data and the synthetic route [17], the **imp-12** could be produced due to excess 4, 7-dichloro-quinoline. As a consequence, the proposed structure of **imp-12** was deduced as 1, 4-bis-(4, 7-dichloroquinoline) piperazine.

The **imp-11** (Figure 3) had the same molecular weight as piperaquine with the accurate protonated ion at m/z 535.2146. The main fragmentation pattern at m/z 288, 260 and 217, was identical to that of piperaquine. It could be concluded that the chlorine atom was substituted on C-5 position of a quinoline ring of **imp-11**, instead of C-7 position of a quinoline ring of piperaquine.

The **imp-6** (Figure 4) with the accurate [M + H]^+^ ion at m/z 551.2094, which was 16 Da higher than that of piperaquine, showed the corresponding protonated formula of C_29_H_33_Cl_2_N_6_O^+^ by TOF-MS. The main fragment ions at 304 and 288 were consistent with those of piperaquine. Based on consideration of the formation of fragment ions and the synthetic route of piperaquine, it was inferred that the probable structure of **imp-6** was proposed as a piperaquine oxygenate with a chloro quinoline ring substitued by hydroxyl group or a piperazine ring of nitrogen oxides. The **imp-8** and **imp-10** (Figure 4) had the same molecular weight and the same fragmentation as **imp-6**, which indicated these two impurities could also be a piperaquine oxygenate. Considering the chromatographic retentions and the hydrophobic characters of three impurities, the probable structures of **imp-6**, **imp-8** and **imp-10** were piperaquine oxygenates with a piperazine ring of nitrogen oxides, C-8 and C-5 positions of a chloro quinoline ring substituted by hydroxyl groups, respectively, which was further confirmed by NMR data (Table 1).

**Table 1:** ^**1**^ **H NMR data of related impurity 6 of piperaquine phosphate**

Position ^a^	Number of protons	Proton chemical shift, δ _H_	*J* ^*b*^(/Hz)
1, 1′	-	-	-
2, 2′	2	8.88	8.45, d
3, 3′	2	7.48	6.35, d
4, 4′	-	-	-
5, 5′	2	8.20	8.33, d
6, 6′	2	7.77	9.0, d
7, 7′	-	-	-
8, 8′	2	8.27	6.36, d
9, 9′	-	-	-
10, 10′	-	-	-
11	-	-	-
12	4	4.35 ~ 4.37	m
13	4	4.26 ~ 4.28	m
14	-	-	-
15	2	3.71	br
16	2	2.73	br
17	2	3.46	br
18	-	-	-
19	4	4.07 ~ 4.12	m
20	4	4.16 ~ 4.21	m

^a^Refer the structural formula in Figure 6 for numbering.

^b^1H-1H coupling constants.

The **imp-7** (Figure 4) with the accurate protonated ion at m/z 585.2160, which was 50 Da higher than that of piperaquine, indicated that piperaquine was replaced by three hydroxyl groups with a piperazine ring opening. According to the main fragment ions at m/z 374 and 288, and the synthetic route of piperaquine, it was inferred that the structure of **imp-7** could also be a piperaquine oxygenate. The probable fragmentation pathways of **imp-7** were shown in Figure 4.

For the **imp-9** (Figure 4), the accurate [M + H]^+^ ion at m/z 501.2536 was 34 Da less than that of piperaquine, suggested a chloro quinoline ring of piperaquine could lose a chlorine atom. A most abundant ion at m/z 288 was also observed in the MS^2^ spectrum of piperaquine. The other main fragment ions at m/z 254, 266 and 211 were consistent with those of piperaquine without a chlorine atom. Thus, the probable structure of the **imp-9** could be 1-(1-4-quinolyl-4-piperazinyl)-3-(1-7-chloro-4-quinolyl-4-piperazinyl) propane. The ESI-MS and TOF-MS data and probable fragmentation pathways of related impurities in piperaquine bulk drug are shown in Figures 2, 3, 4 and 5.

### Structure confirmation of impurities by ^1^H and ^13^C NMR

**Figure 6:**
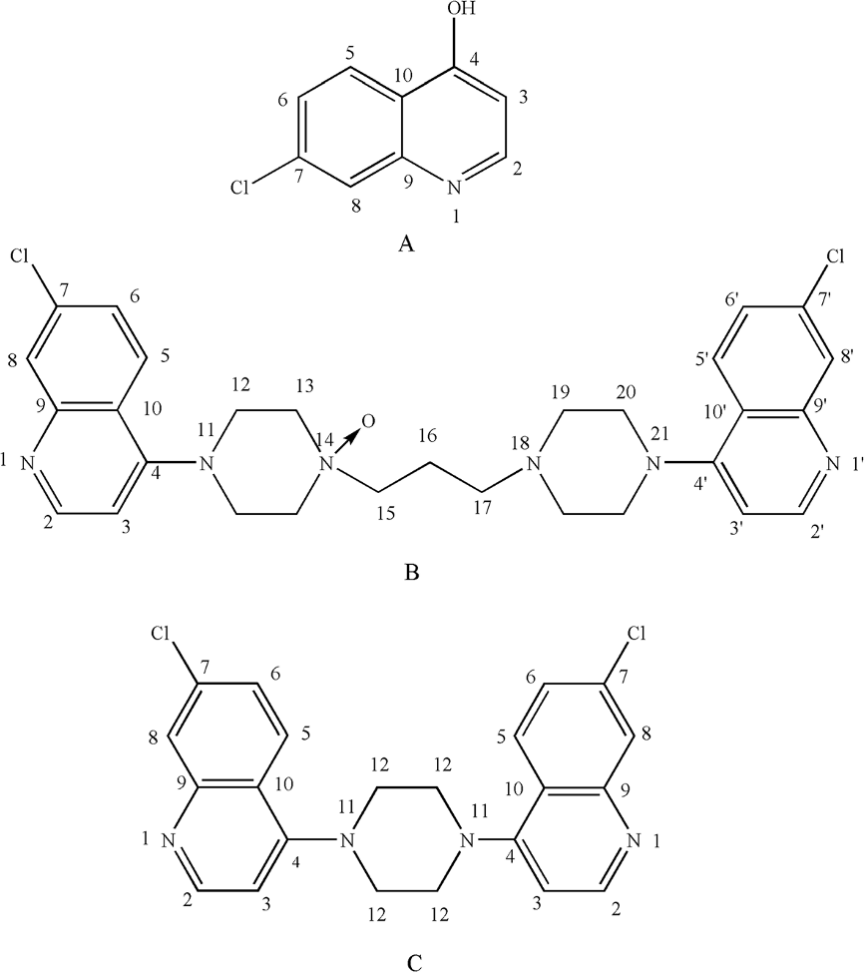
**NMR assignments of (A) imp-2, (B) imp-6 and (C) imp-12.**

The TOF-MS spectrum of **imp-2** had the accurate [M + H]^+^ ion at m/z 180.0207, consistent with the molecular formula of C_9_H_6_ClNO. In the solution of DMSO-d6 and TFA-d, the ^1^H-NMR data showed six hydrogen signals with the area ratio of 1:1:1:1:1:1. By the experiment of D_2_O exchange, the chemical shift of active hydrogen had a downfield shift from 11.9 ppm to 3.5 ppm, and other five hydrogen atoms were located at aromatic heterocyclic rings. The ^13^C NMR and DEPT spectrum showed the presence of nine carbon signals including four quaternary carbons and five tertiary carbons. The ^1^H-NMR spectrum showed that the presence of quinoline ring signals [δ_H_ 7.95 (1H, d, *J* = 7.4 Hz, H-2), δ_H_ 6.10 (1H, d, *J* = 7.4 Hz, H-3), δ_H_ 11.91 (1H, s, H-4), δ_H_ 8.11 (1H, d, *J* = 8.7 Hz, H-5), δ_H_ 7.33 (1H, d, *J* = 10 Hz, H-6) and δ_H_ 7.63 (1H, s, H-8)]. With the aid of 1H-1H COSY, HMQC and HMBC spectrums, all the ^1^H and ^13^C NMR data of **imp-2** were listed in Table 2. The ^13^C NMR and HMBC spectrums showed the presence of four quaternary carbon atoms [δ_C_ 176.2 (C-4), δ_C_ 136.2 (C-7), δ_C_ 140.8 (C-9) and δ_C_ 124.3 (C-10), δ_H_ 6.10 (1H, d, *J* = 7.4 Hz, H-3) and δ_H_ 11.91 (1 H, s, H-4). Hence, the chemical structure of **imp-2** was verified as shown in Figure 6.

**Table 2:** ^**1**^ **H,** ^**13**^ **C NMR data of related impurity (2 and 12) of piperaquine phosphate**

	2		12	
Position ^a^	δ _H_ (*J* ^b^/Hz)	δ _C_	δ _H_ (*J* ^*b*^/Hz)	δ _C_
1	-	-	-	-
2	7.95 (1H, d, 7.4)	139.9	8.45 (2H, d, 7.1)	144.5
3	6.10 (1H, d, 7.4)	109.2	7.09 (2H, d, 7.2)	107.2
4	11.91 (1H, s)	176.2	-	164.2
5	8.11 (1H, d, 8.7)	127.2	8.33 (2H, d, 9.3)	131.4
6	7.33 (1H, d, 10)	123.4	7.71 (2H, d, 7.8)	131.0
7	-	136.2	-	121.7
8	7.63 (1H, s)	117.4	8.07 (2H, d, 1.7)	122.8
9	-	140.8	-	143.4
10	-	124.3	-	120.4
11	-	-	-	-
12	-	-	4.48 (8H, s)	52.9

^a^Refer the structural formula in Figure 6 for numbering.

^b^1H-1H coupling constants.

The TOF-MS and ESI-MS analysis of **imp-6** provided a molecular formula of C_29_H_32_Cl_2_N_6_O ([M + H]^+^, m/z 551.2094). The ^1^H-NMR and ^1^H-^1^H COSY spectrums showed that the presence of quinoline ring signals [δ_H_ 8.27 (2H, d, *J* =25.8 Hz, H-8, 8′), δ_H_ 8.88 (2H, d, *J* = 6.35 Hz, H-2, 2′), δ_H_ 7.77 (2H, d, *J* = 9 Hz, H-6, 6′) and δ_H_ 7.48 (2H, d, *J* = 6.4 Hz, H-3, 3′). The presence of eight methylene group signals [δ_H_ 4.34 ~ 4.37 (4H, m, H-12), δ_H_ 4.26 ~ 4.28 (4H, m, H13), δ_H_ 4.07 ~ 4.12 (4H, m, H-19) and δ_H_ 4.16 ~ 4.21 (4H, m, H-20)], indicated that these methylene groups were located at two piperazine rings. Furthermore, three methylene group signals [δ_H_ 3.71 (2H, br, H-15), δ_H_ 2.73 (2H, br, H-16) and δ_H_ 3.46 (2H, br, H-17)] were observed, with significant correlation in the ^1^H-^1^H COSY spectrum, and H-15 had an downfield shift due to oxidation at C-14. Thus, **imp-6** was characterized as shown in Figure 6.

As to **imp-12**, ESI-MS and TOF-MS data showed the accurate protonated ion at m/z 409.0987, with a molecular formula of C_22_H_18_Cl_2_N_4_. The ^1^H and ^13^C NMR data of **imp-12** were in agreement with the literature data [17], thus the structure of **imp-12** was confirmed as 1, 4-bis-(4, 7-dichoroquinoline) piperazine (Figure 6).

All ^1^H and ^13^C NMR signals of **imp-2**, **6** and **12** were assigned in Tables 1 and 2, and all the structure deductions were also confirmed by ESI-MS and TOF-MS.

### Formation of impurity

In the HPLC-UV/DAD, ESI-MS and TOF-MS experiments, twelve related impurities were detected in piperaquine phosphate bulk drug. The starting material for piperaquine phosphate, i.e. 4, 7-dichloro-quinoline, was confirmed by the literature [17], and the **imp-1, 2, 4** were the isomers of 4, 7-dichloro-quinoline bulk. According to the synthetic route, the **imp-3, 5, 11, 12** were the by-product in the synthetic reaction of piperaquine phosphate. Based on the experiment of forced degradation samples, the **imp-6**–**8**, **10** were the oxidation products and the **imp-9** was the degradation product of piperaquine phosphate. The possible mechanism of formation of impurities was depicted in Figure 5.

### ***In silico*toxicological predictions o**f **impurities**

To evaluate general toxicological and carcinogenic alerts for the related impurities of piperaquine *in silico*, Toxtree^®^ and Derek^®^, the knowledge-based expert systems, were used from different two (Q)SAR programs. Since the **imp-2, 6, 12** were the main related impurities in piperaquine bulk, the toxicity profiles of three impurities were of paramount importance. By the module of the Cramer rules with extensions in Toxtree^®^, the **imp-2, 6, 12** were predicted to general toxicity risks (class III). Based on Benigni/Bossa rulebase for mutagenicity and carcinogenicity, three related impurities were predicted negative for carcinogenicity (genotox and nogenotox) and mutagenicity. The predicted results of the module of structure alerts for the *in vivo* micronucleus assay in rodents, showed that the **imp-6** and **12** were all H-acceptor-path3-H-acceptors except the **imp-2**. Derek^®^ predicted several toxicity alerts for the **imp 2, 6, 12**: carcinogenicity in mammal (**imp-2**), hERG channel inhibition (**imp-6** and **12**), hepatotoxicity (**imp-6** and **12**), mutagenicity (**imp-2**) and alpha-2-mu-Globulin nephropathy (**imp-12**).

The other piperaquine related impurities were also predicted by Toxtree^®^ to have a high general toxicity risks similar with the **imp-2, 6, 12** (class III). Furthermore, the **imp-1**, **3**, **5** and **7–11** were predicted negative for carcinogenicity (genotox and nogenotox) and mutagenicity, while the **imp-4** structure of primary aromatic amine led to structural alert for genotoxic carcinogenicity. From the module of structure alerts for the *in vivo* micronucleus assay in rodents, the **imp-5** and **7**–**11** were also H-acceptor-path3-H-acceptors, due to the similar structures with the **imp-6** and **12**. The prediction results of Derek^®^ indicated a limit toxicity profile for other impurities, such as carcinogenicity for the **imp-8**, hERG channel inhibition for the **imp-3**–**6** and **imp-8**–**11**, hepatotoxicity for the **imp-4, 6, 7, 9–11**, skin sensitization for the **imp-1, 8** and alpha-2-mu-Globulin nephropathy for the **imp-4, 7–11**. Only the **imp-3** showed a non-toxic prediction compared to other impurities.

According to the toxicological concern, the daily dosage of compounds classified in class III should be below 90 μg/person (60 kg)/day to be validated as non toxic [18]. Therefore, the toxicity predicts of **imp-1**–**12** provide valuable data for clinical use of piperaquine dose. In China, the use of the piperaquine preparations is cautioned for pregnant women and patients with severe acute liver, kidney and heart diseases.

## Conclusions

Twelve impurities of piperaquine phosphate bulk drug were detected by HPLC-UV/DAD, ESI-MS and TOF-MS. The structures of impurities were proposed on the basis of ESI-MS and TOF-MS, fragmentation mechanism and synthetic procedure. The **imp-2**, **6** and **12**, three main related impurities, were synthesized or isolated from the oxidation samples of piperaquine phosphate by column chromatography and these structures were confirmed by NMR spectrum. Starting material along with impurities, synthetic by-products, oxidation and degradation were the main sources for the formation of these impurities. The *in-silico* toxicological investigation (Toxtree^®^ and Derek^®^) indicated three main related impurities (**imp-2**, **6** and **12**) had general toxicity risks and nogenotox, which provided the useful data in the research of piperaquine.

## Electronic supplementary material

Additional file 1: **The analytical development process of the piperaquine phosphate related impurities.** (DOC 32 KB)

Additional file 2: **UV chromatogram of piperaquine crude samples from different API suppliers.** A, B, C and D represented the samples from Shanghai Zhongxi Pharmaceutical Factory Co., LTD (ZX1006098, ZX1006097, ZX1006075 and ZX1006074). E, F, G, H and I represented the samples from Chongqing Kangle Pharmaceutical Factory Co., LTD (KL111107-RS, KL111101, KL110401-2, KL110401-1 and KL091101). J and K represented the samples from Chongqing Southwest No.2 Pharmaceutical Factory Co., LTD (XN1206003 and XN1206002). (PDF 892 KB)

Additional file 3: **ESI-MS** ^**n**^ **spectra acquired from [M + H]** ^**+**^ **ions of imp-1.** (PDF 27 KB)

Additional file 4: **ESI-MS** ^**n**^ **spectra acquired from [M + H]** ^**+**^ **ions of imp-2.** (PDF 27 KB)

Additional file 5: **ESI-MS** ^**n**^ **spectra acquired from [M + H]** ^**+**^ **ions of imp-3.** (PDF 26 KB)

Additional file 6: **ESI-MS** ^**n**^ **spectra acquired from [M + H]** ^**+**^ **ions of imp-4.** (PDF 26 KB)

Additional file 7: **ESI-MS** ^**n**^ **spectra acquired from [M + H]** ^**+**^ **ions of imp-5.** (PDF 26 KB)

Additional file 8: **ESI-MS** ^**n**^ **spectra acquired from [M + H]** ^**+**^ **ions of imp-6.** (PDF 27 KB)

Additional file 9: **ESI-MS** ^**n**^ **spectra acquired from [M + H]** ^**+**^ **ions of imp-7.** (PDF 28 KB)

Additional file 10: **ESI-MS** ^**n**^ **spectra acquired from [M + H]** ^**+**^ **ions of imp-8.** (PDF 28 KB)

Additional file 11: **ESI-MS** ^**n**^ **spectra acquired from [M + H]** ^**+**^ **ions of imp-9.** (PDF 28 KB)

Additional file 12: **ESI-MS** ^**n**^ **spectra acquired from [M + H]** ^**+**^ **ions of imp-10.** (PDF 28 KB)

Additional file 13: **ESI-MS** ^**n**^ **spectra acquired from [M + H]** ^**+**^ **ions of imp-11.** (PDF 27 KB)

Additional file 14: **ESI-MS** ^**n**^ **spectra acquired from [M + H]** ^**+**^ **ions of imp-12.** (PDF 27 KB)

Additional file 15: **ESI-MS** ^**n**^ **spectra acquired from [M + H]** ^**+**^ **ions of piperaquine.** (PDF 27 KB)
