# Supplementary material for: Stability profiling of anti-malarial drug piperaquine phosphate and impurities by HPLC-UV, TOF-MS, ESI-MS and NMR
Source: Malar J. 2014 Oct 13;13:401. doi: 10.1186/1475-2875-13-401 (PMC4210591; doi:10.1186/1475-2875-13-401)
Supplement: Supplementary file 1 — Additional file 1: The analytical development process of the piperaquine phosphate related impurities. (DOC 32 KB) [file 12936_2014_3569_MOESM1_ESM.doc]

## Additional file 1 The analytical development process of the piperaquine phosphate related impurities.

| Mobile phase system | Isocratic/ Gradient Elution  *T* (min)/ % Acetonitrile | Results |
| --- | --- | --- |
| A: Phosphoric acid solution; B: 0.1% trifluoroacetic acid;  C: Acetonitrile  (Chinese Pharmacopoeia) | 0.035:80:20 | The Retention time of piperaquine was 7.7 min with baseline separation of impurities, but the separation of the impurities was not achieved. |
| A: 0.2% formic acid solution; B:acetonitrile | 0/6, 3/6, 28/20,  45/50, 45.1/6, 55/6 | The Retention time of piperaquine was 8.9 min with baseline separation of impurities, but the separation of the impurities was not achieved. |
| A: 0.01 M ammonium acetate solution with pH of 7.0;  B:acetonitrile | 0/40, 6/40, 55/90,  55.1/40, 60/40 | The Retention time of piperaquine was 33.6 min with baseline separation of impurities, but the separation of the impurities was not achieved. |
| A: 0.1% ammonium acetate solution with pH of 7.0;  B:acetonitrile | 0/40, 5/40, 54/100,  54.1/40, 60/40 | The Retention time of piperaquine was 30.98 min with baseline separation of impurities, and the baseline separation of the impurities was also achieved. |
