# Supplementary figures and images for: Stability profiling of anti-malarial drug piperaquine phosphate and impurities by HPLC-UV, TOF-MS, ESI-MS and NMR
Source: Malar J. 2014 Oct 13;13:401. doi: 10.1186/1475-2875-13-401 (PMC4210591; doi:10.1186/1475-2875-13-401)

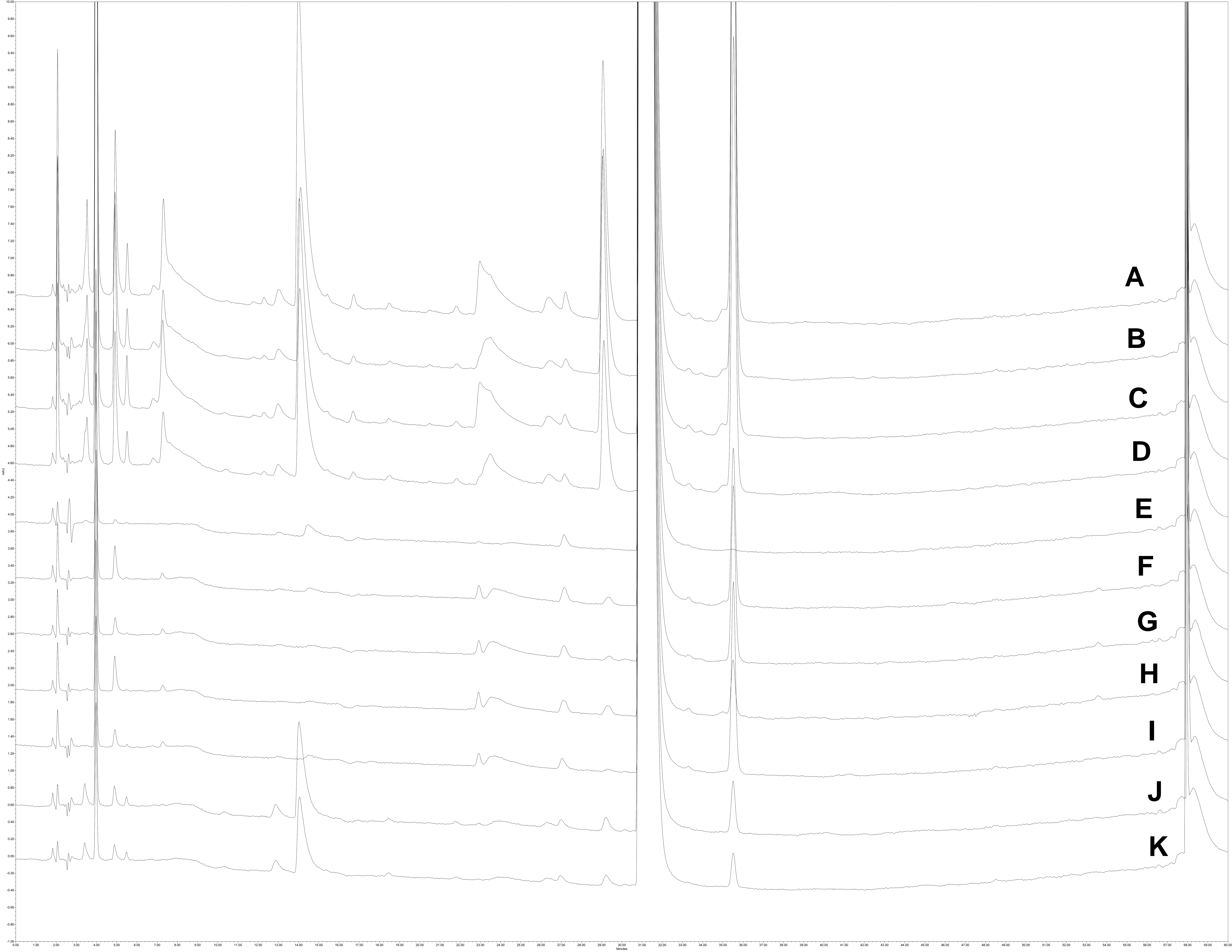

Supplement: Supplementary file 2 — Additional file 2: UV chromatogram of piperaquine crude samples from different API suppliers. A, B, C and D represented the samples from Shanghai Zhongxi Pharmaceutical Factory Co., LTD (ZX1006098, ZX1006097, ZX1006075 and ZX1006074). E, F, G, H and I represented the samples from Chongqing Kangle Pharmaceutical Factory Co., LTD (KL111107-RS, KL111101, KL110401-2, KL110401-1 and KL091101). J and K represented the samples from Chongqing Southwest No.2 Pharmaceutical Factory Co., LTD (XN1206003 and XN1206002). (PDF 892 KB) [file 12936_2014_3569_MOESM2_ESM.pdf]

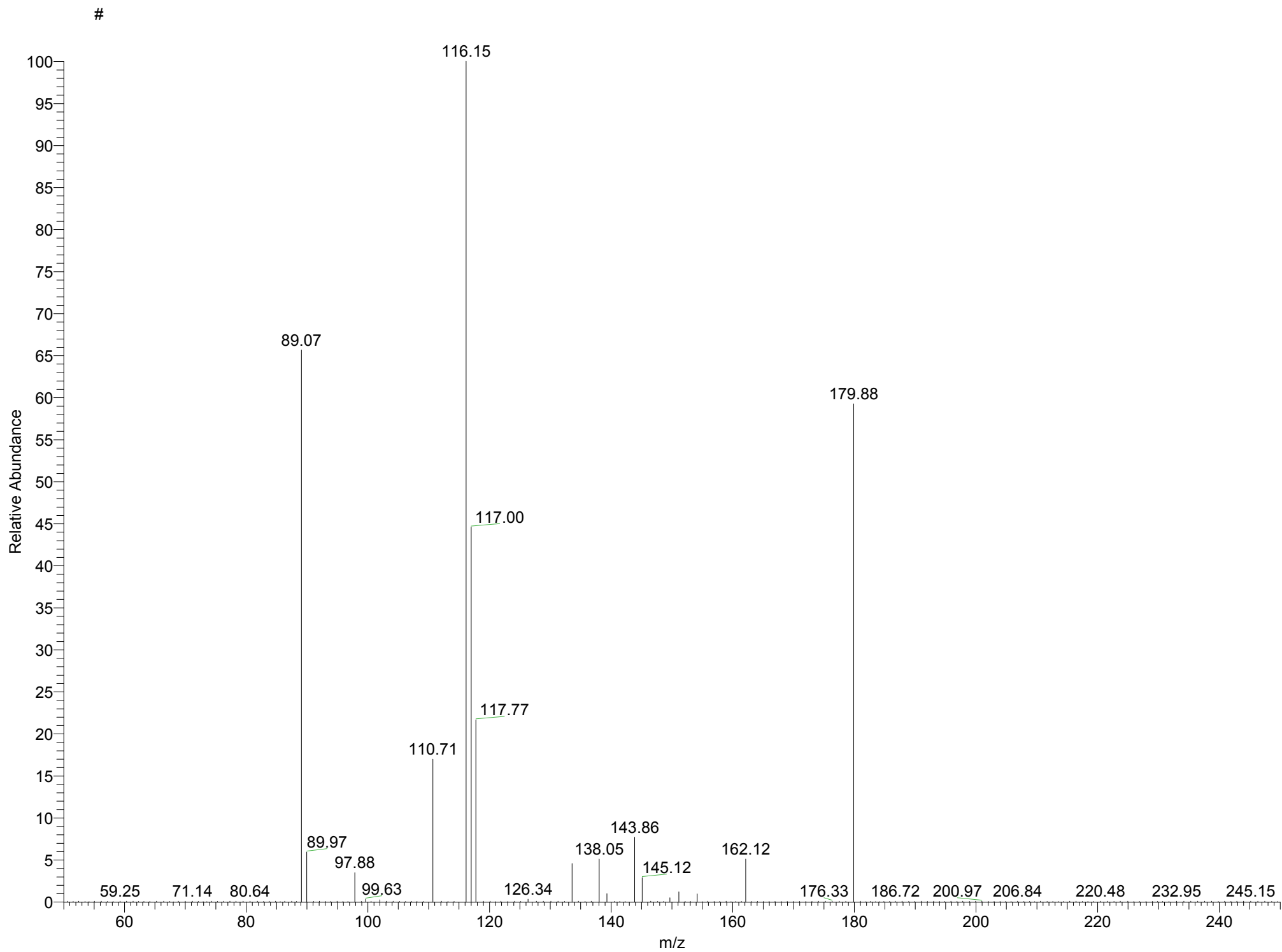

Supplement: Supplementary file 3 — Additional file 3: ESI-MS n spectra acquired from [M + H] + ions of imp-1. (PDF 27 KB) [file 12936_2014_3569_MOESM3_ESM.pdf]

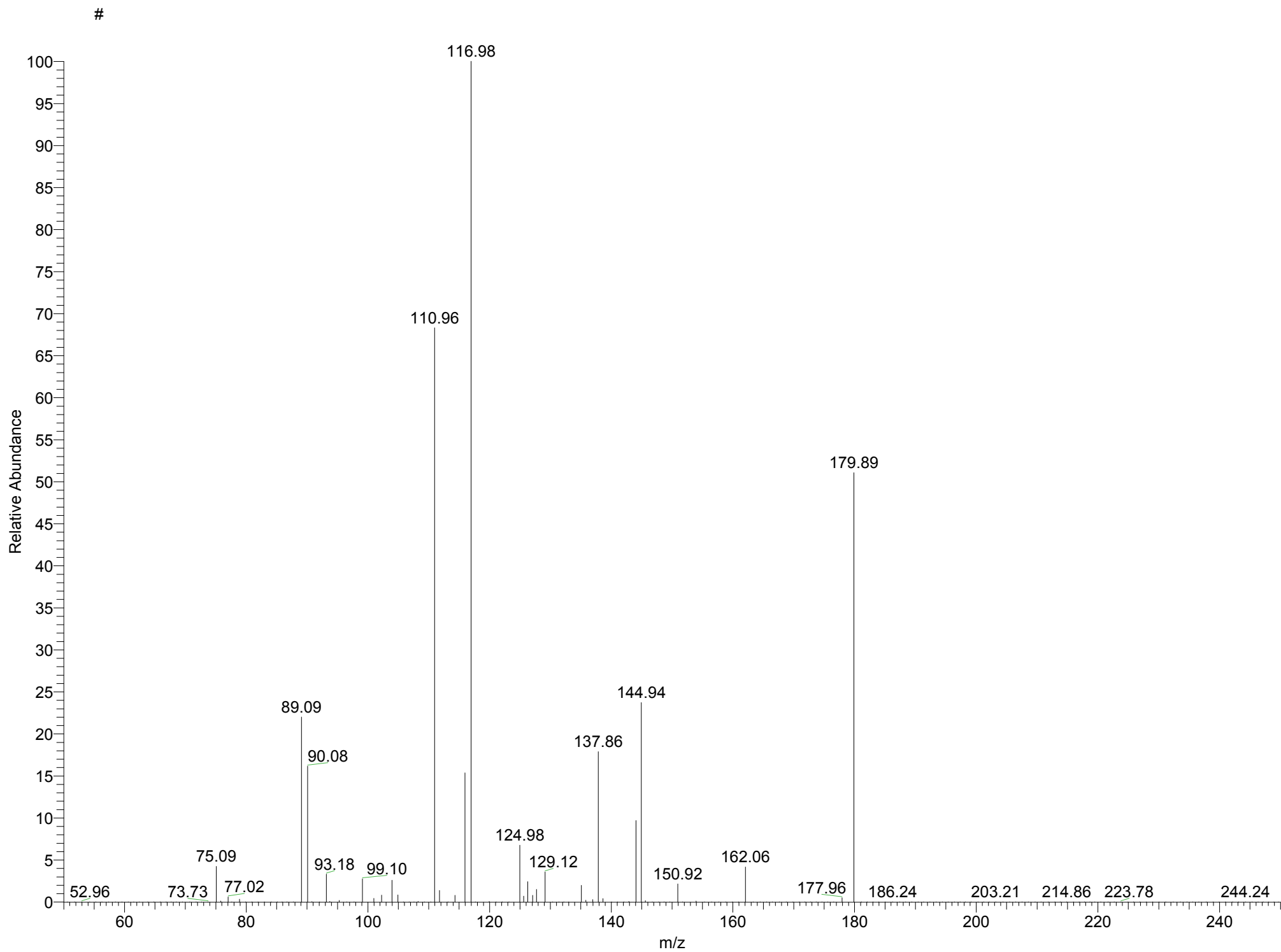

Supplement: Supplementary file 4 — Additional file 4: ESI-MS n spectra acquired from [M + H] + ions of imp-2. (PDF 27 KB) [file 12936_2014_3569_MOESM4_ESM.pdf]

#

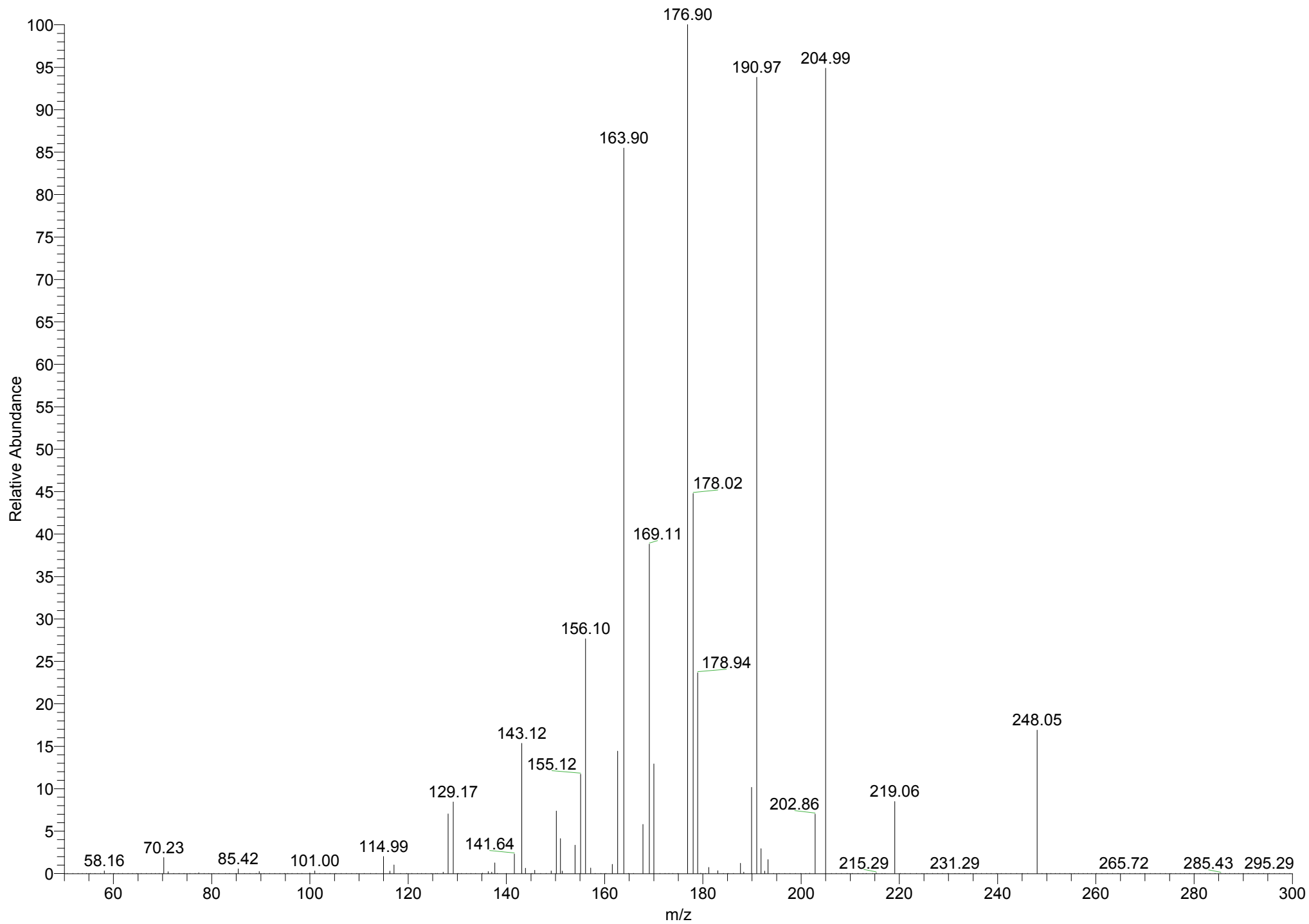

Supplement: Supplementary file 5 — Additional file 5: ESI-MS n spectra acquired from [M + H] + ions of imp-3. (PDF 26 KB) [file 12936_2014_3569_MOESM5_ESM.pdf]

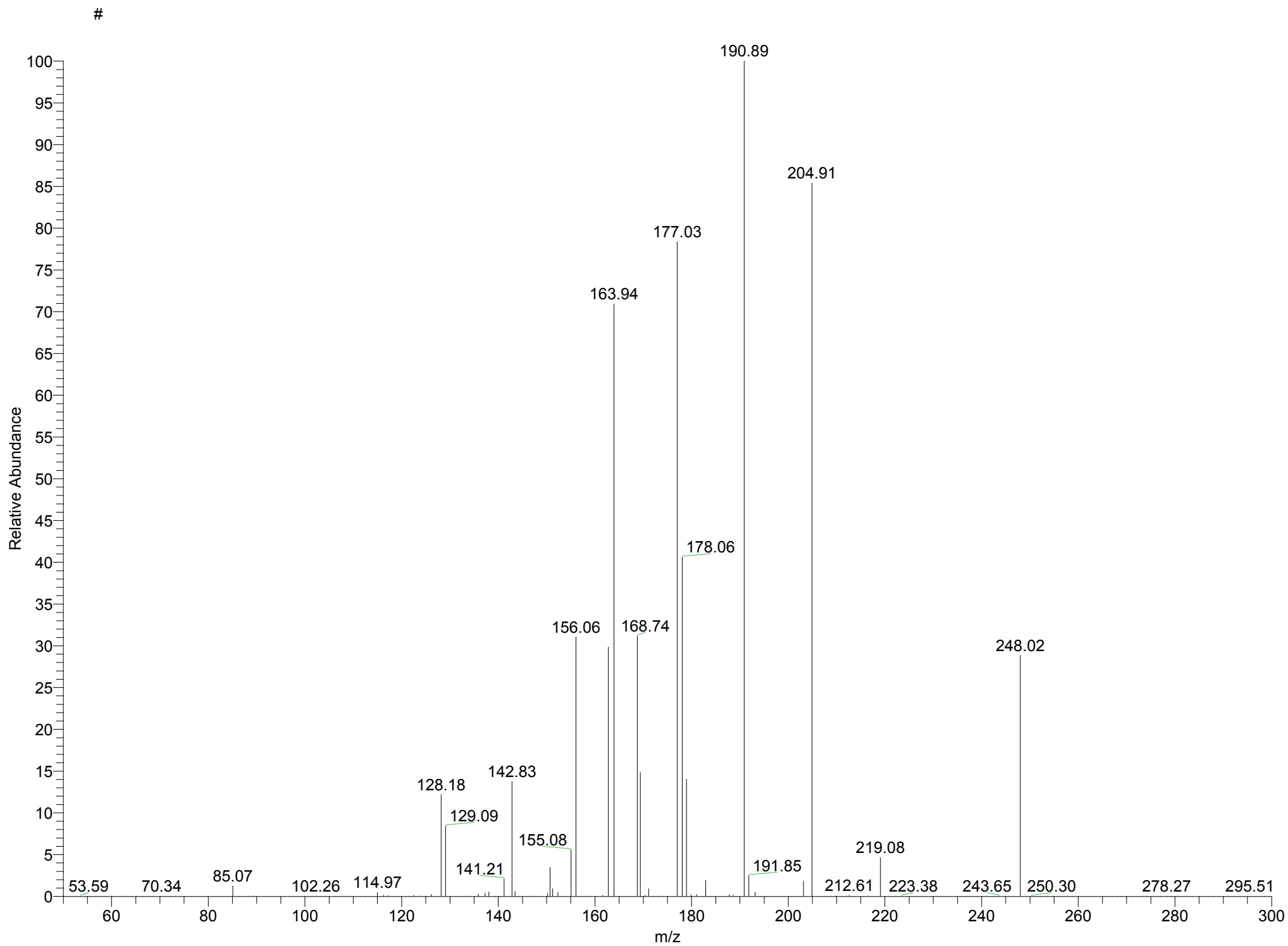

Supplement: Supplementary file 6 — Additional file 6: ESI-MS n spectra acquired from [M + H] + ions of imp-4. (PDF 26 KB) [file 12936_2014_3569_MOESM6_ESM.pdf]

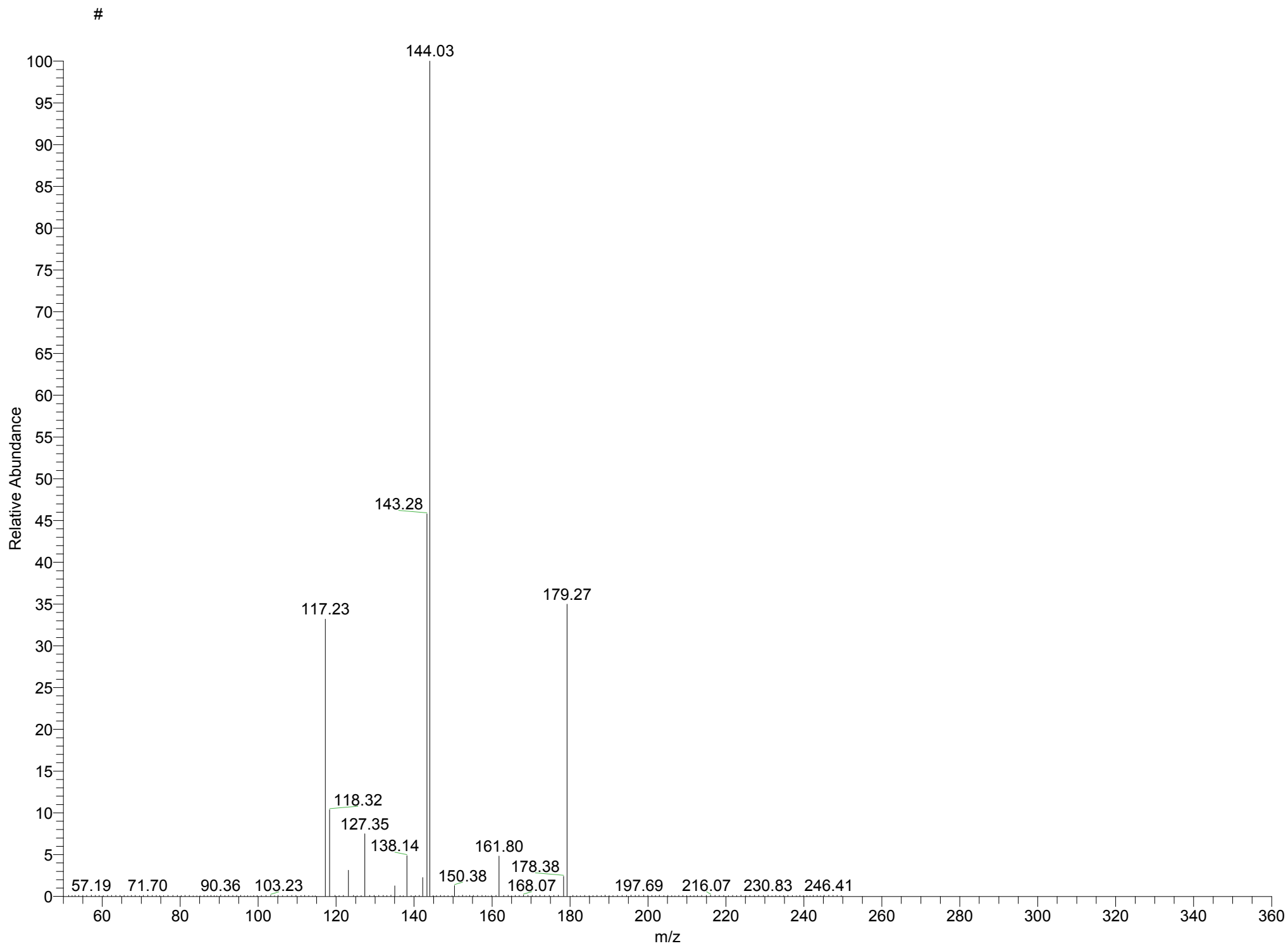

Supplement: Supplementary file 7 — Additional file 7: ESI-MS n spectra acquired from [M + H] + ions of imp-5. (PDF 26 KB) [file 12936_2014_3569_MOESM7_ESM.pdf]

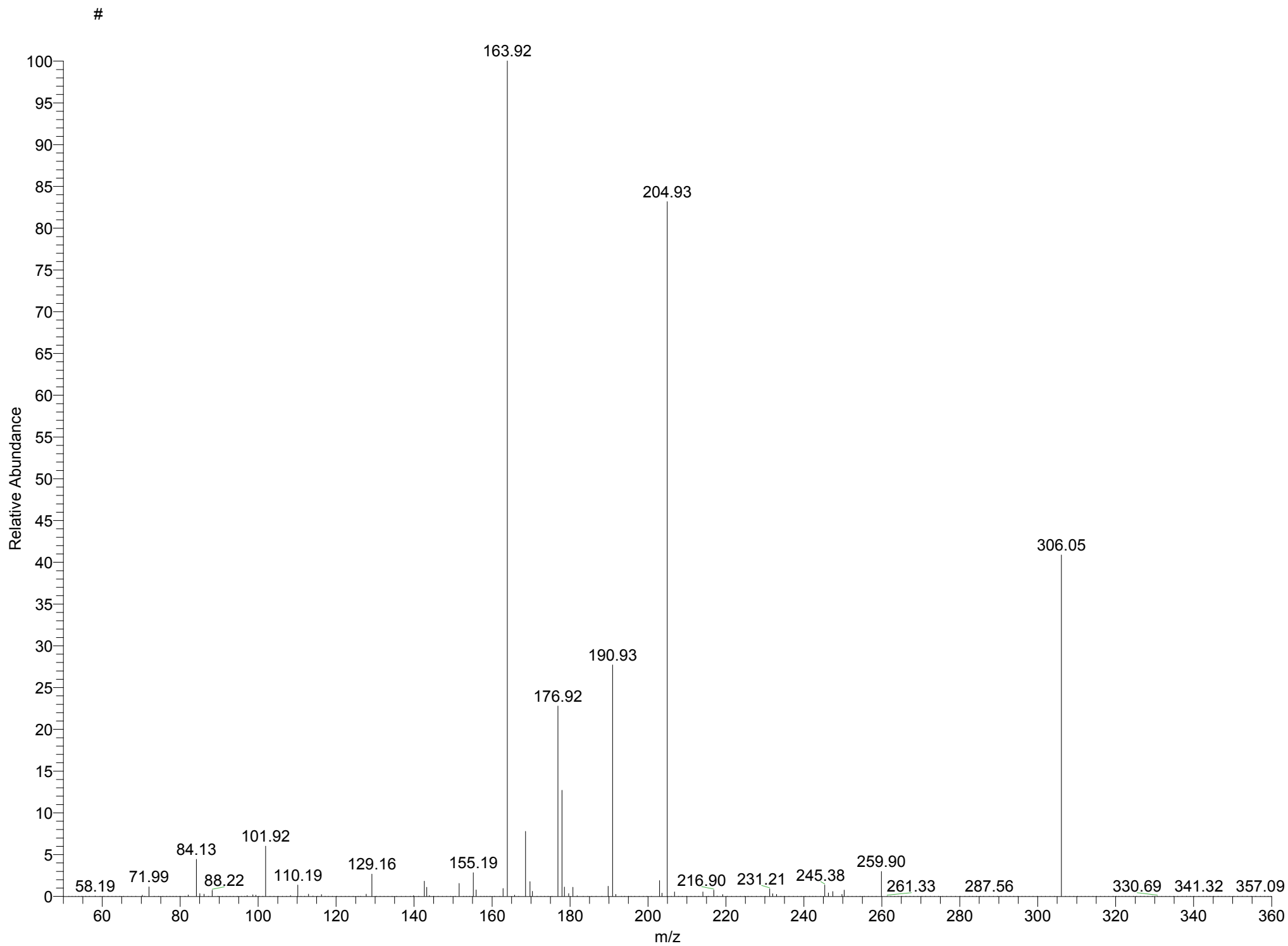

Supplement: Supplementary file 8 — Additional file 8: ESI-MS n spectra acquired from [M + H] + ions of imp-6. (PDF 27 KB) [file 12936_2014_3569_MOESM8_ESM.pdf]

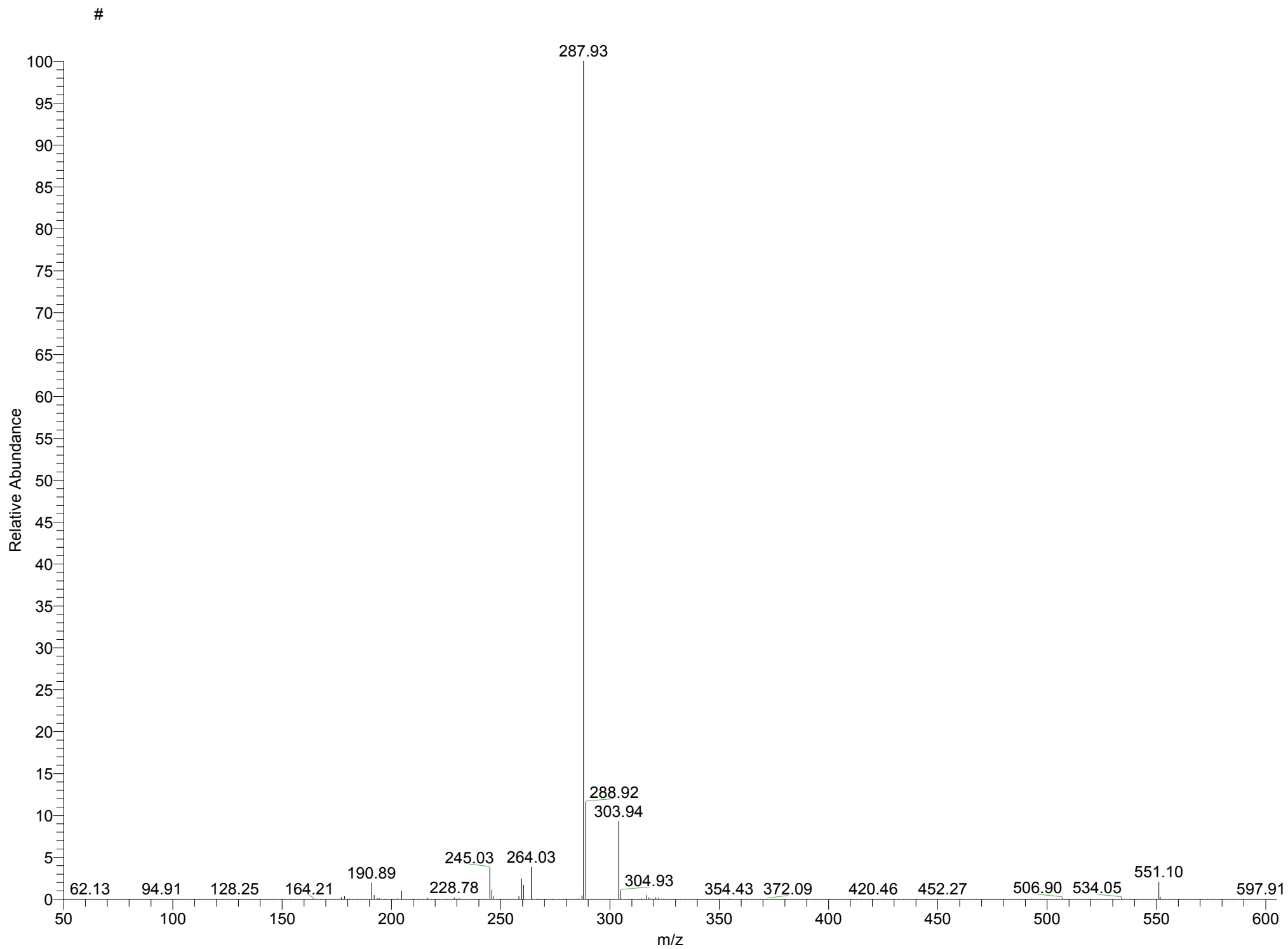

Supplement: Supplementary file 9 — Additional file 9: ESI-MS n spectra acquired from [M + H] + ions of imp-7. (PDF 28 KB) [file 12936_2014_3569_MOESM9_ESM.pdf]

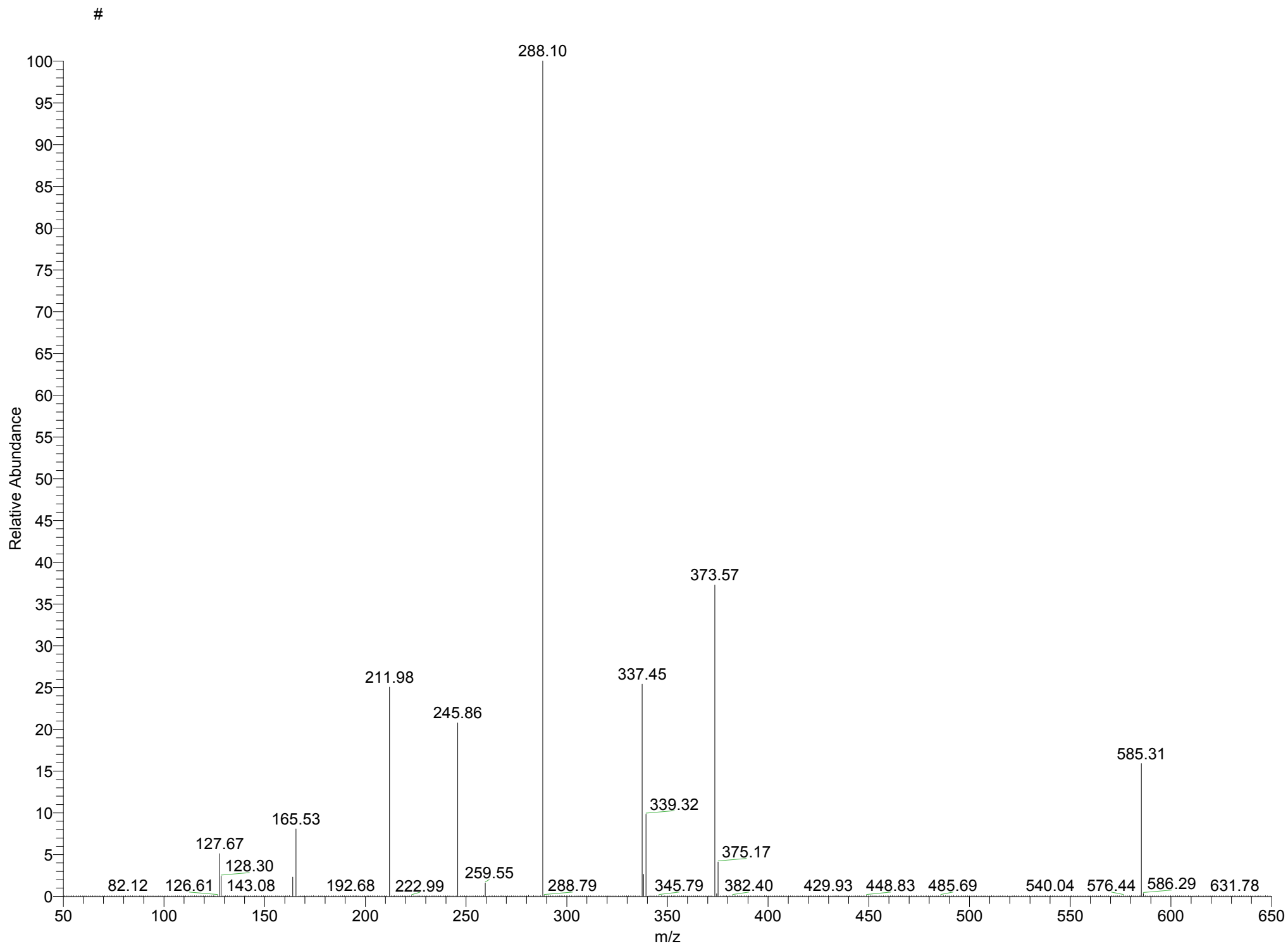

Supplement: Supplementary file 10 — Additional file 10: ESI-MS n spectra acquired from [M + H] + ions of imp-8. (PDF 28 KB) [file 12936_2014_3569_MOESM10_ESM.pdf]

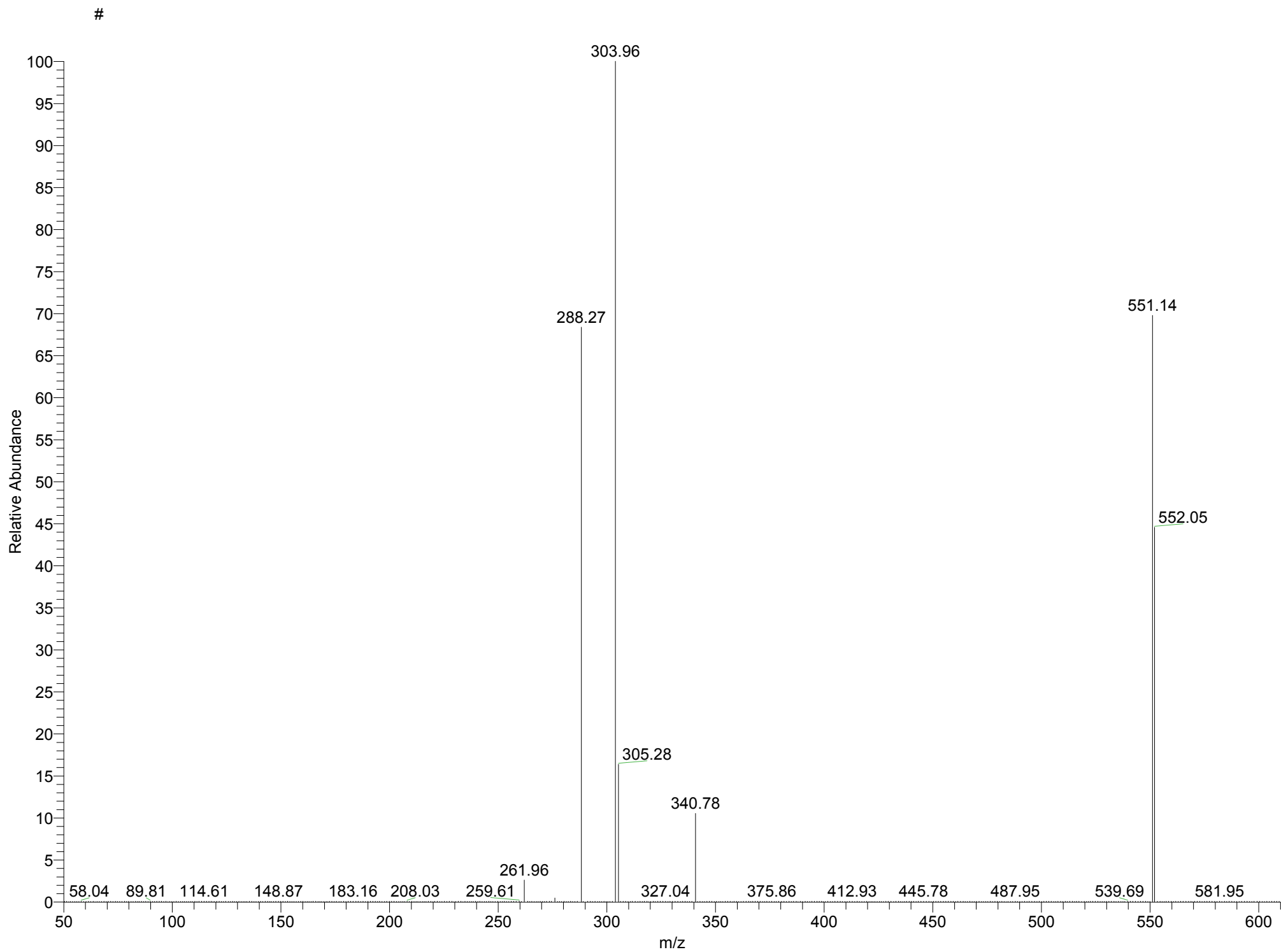

Supplement: Supplementary file 11 — Additional file 11: ESI-MS n spectra acquired from [M + H] + ions of imp-9. (PDF 28 KB) [file 12936_2014_3569_MOESM11_ESM.pdf]

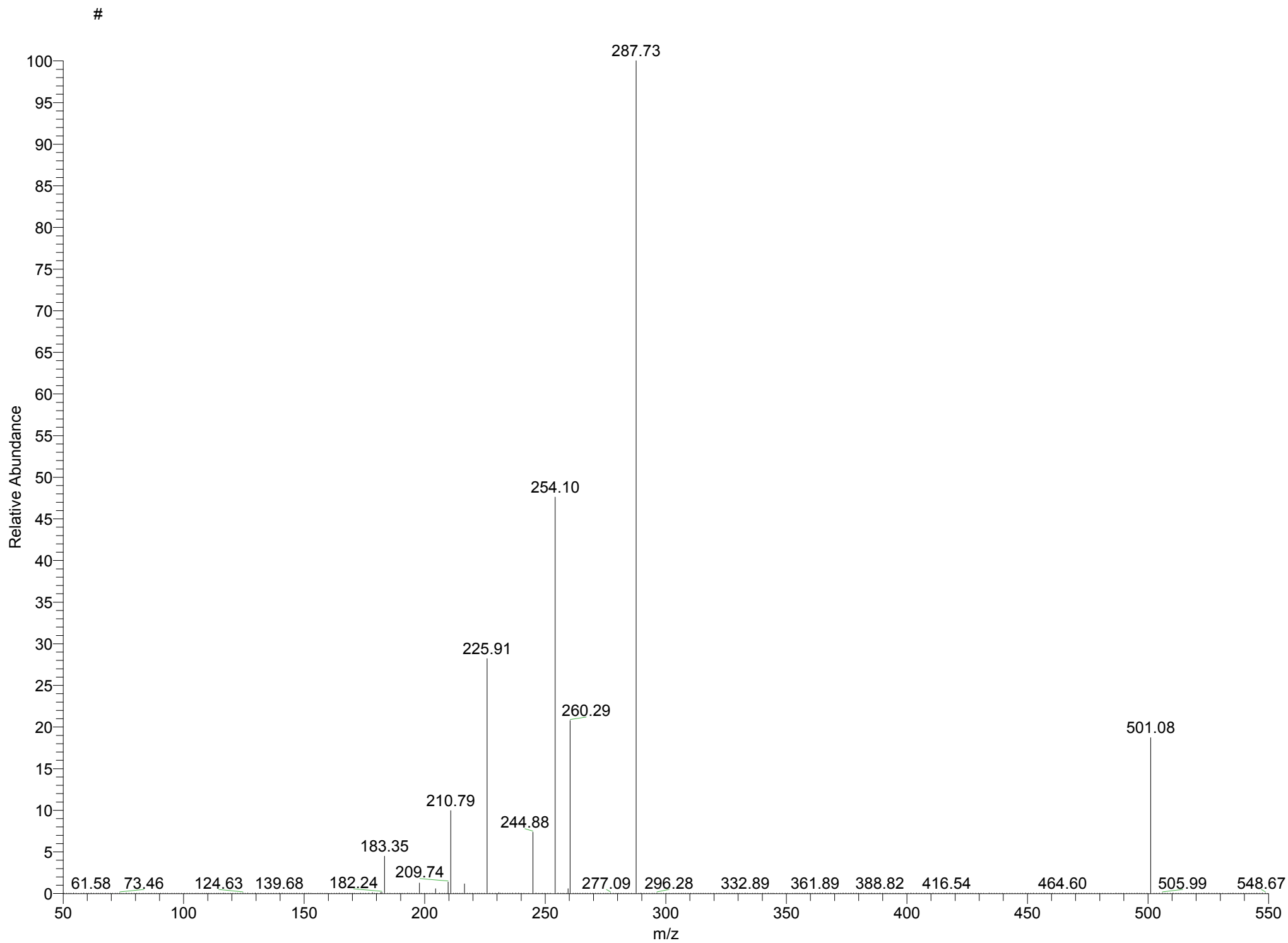

Supplement: Supplementary file 12 — Additional file 12: ESI-MS n spectra acquired from [M + H] + ions of imp-10. (PDF 28 KB) [file 12936_2014_3569_MOESM12_ESM.pdf]

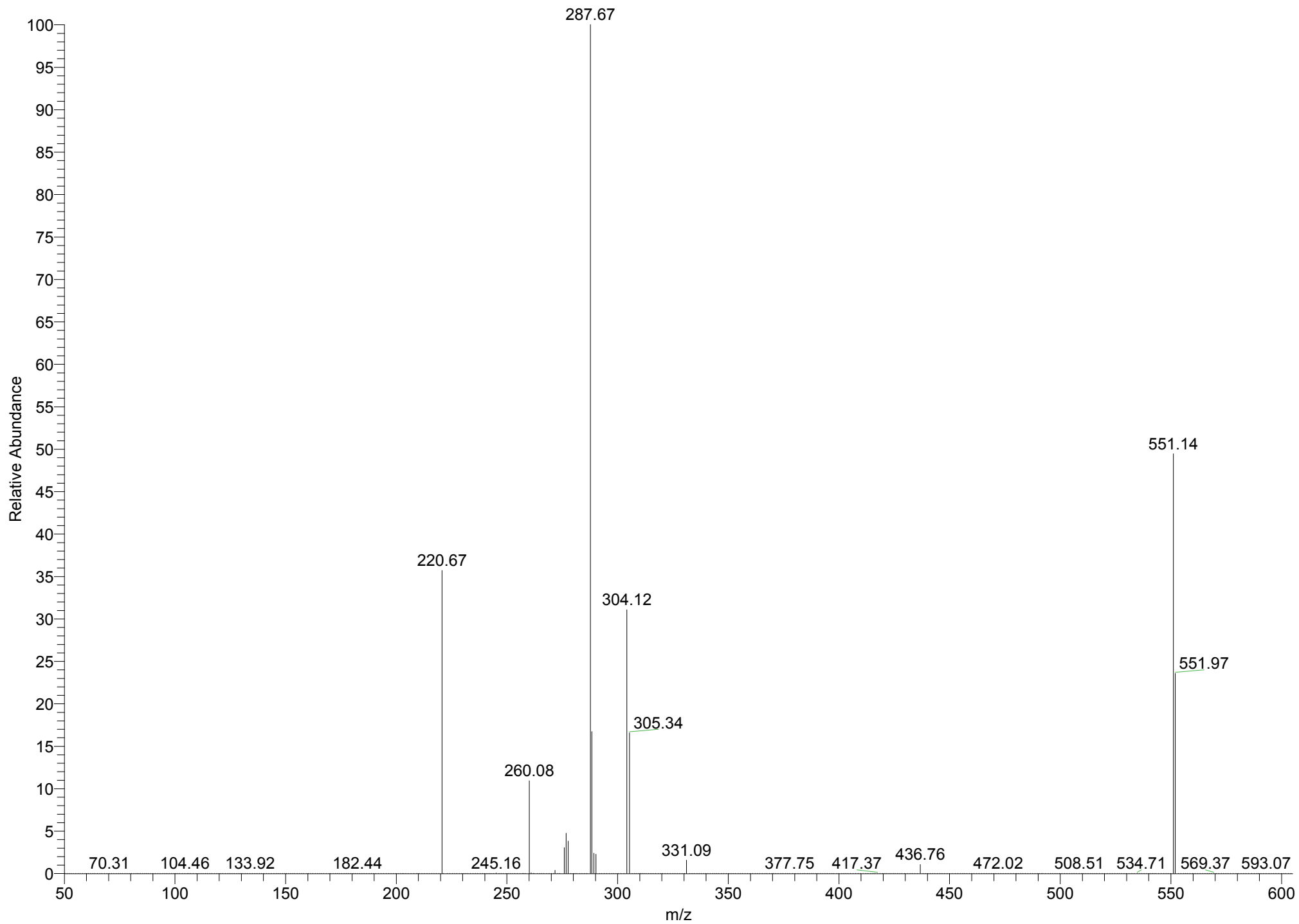

Supplement: Supplementary file 13 — Additional file 13: ESI-MS n spectra acquired from [M + H] + ions of imp-11. (PDF 27 KB) [file 12936_2014_3569_MOESM13_ESM.pdf]

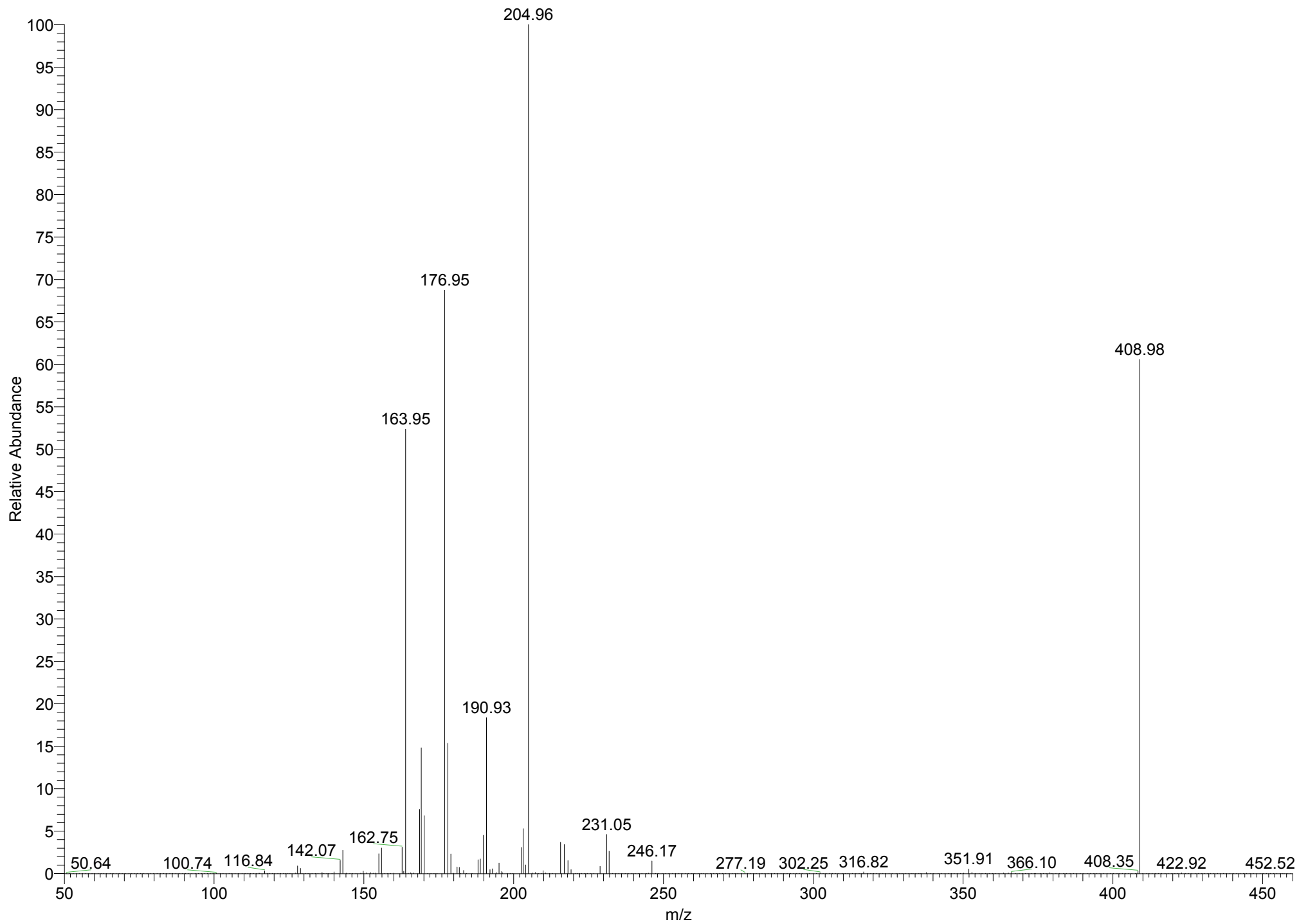

Supplement: Supplementary file 14 — Additional file 14: ESI-MS n spectra acquired from [M + H] + ions of imp-12. (PDF 27 KB) [file 12936_2014_3569_MOESM14_ESM.pdf]

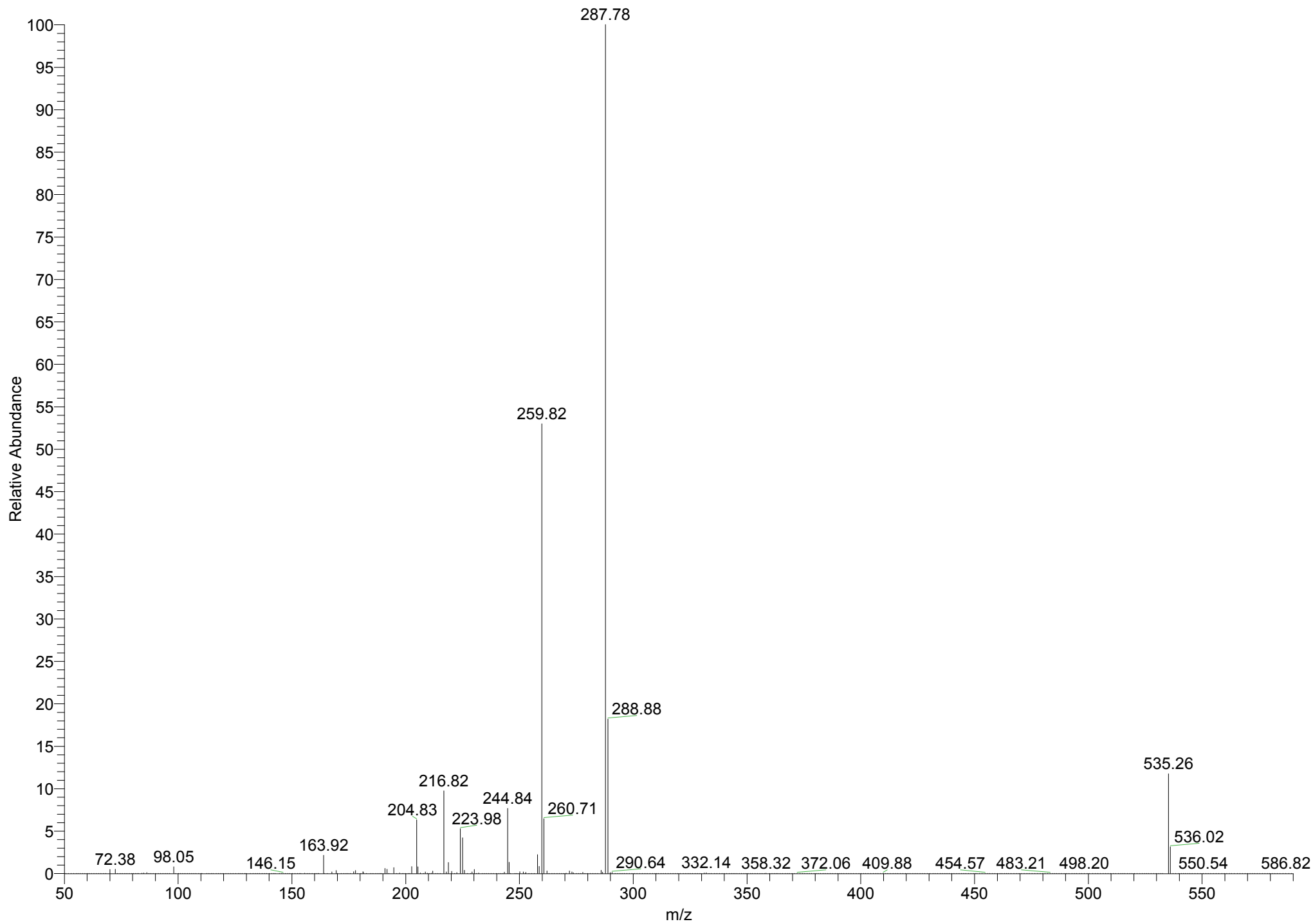

Supplement: Supplementary file 15 — Additional file 15: ESI-MS n spectra acquired from [M + H] + ions of piperaquine. (PDF 27 KB) [file 12936_2014_3569_MOESM15_ESM.pdf]
